# Investigating Additive and Replacing Horizontal Gene Transfers Using Phylogenies and Whole Genomes

**DOI:** 10.1093/gbe/evae180

**Published:** 2024-08-20

**Authors:** Lina Kloub, Sophia Gosselin, Joerg Graf, Johann Peter Gogarten, Mukul S Bansal

**Affiliations:** School of Computing, University of Connecticut, 371 Fairfield Way, Unit 4155, Storrs, CT 06269-4155, USA; Department of Molecular and Cell Biology, University of Connecticut, 91 North Eagleville Road, Unit 3125, Storrs, CT 06269-3125, USA; Department of Molecular and Cell Biology, University of Connecticut, 91 North Eagleville Road, Unit 3125, Storrs, CT 06269-3125, USA; Pacific Biosciences Research Center, University of Hawaii, Honolulu, HI 96822, USA; Department of Molecular and Cell Biology, University of Connecticut, 91 North Eagleville Road, Unit 3125, Storrs, CT 06269-3125, USA; The Institute for Systems Genomics, University of Connecticut, Storrs, CT 06269, USA; School of Computing, University of Connecticut, 371 Fairfield Way, Unit 4155, Storrs, CT 06269-4155, USA; The Institute for Systems Genomics, University of Connecticut, Storrs, CT 06269, USA

**Keywords:** horizontal gene transfer, additive transfer, replacing transfer, prokaryotes, *Aeromonas*, genome evolution

## Abstract

Horizontal gene transfer (HGT) is fundamental to microbial evolution and adaptation. When a gene is horizontally transferred, it may either add itself as a new gene to the recipient genome (possibly displacing nonhomologous genes) or replace an existing homologous gene. Currently, studies do not usually distinguish between “additive” and “replacing” HGTs, and their relative frequencies, integration mechanisms, and specific roles in microbial evolution are poorly understood. In this work, we develop a novel computational framework for large-scale classification of HGTs as either additive or replacing. Our framework leverages recently developed phylogenetic approaches for HGT detection and classifies HGTs inferred between terminal edges based on gene orderings along genomes and phylogenetic relationships between the microbial species under consideration. The resulting method, called *DART*, is highly customizable and scalable and can classify a large fraction of inferred HGTs with high confidence and statistical support. Our application of DART to a large dataset of thousands of gene families from 103 *Aeromonas* genomes provides insights into the relative frequencies, functional biases, and integration mechanisms of additive and replacing HGTs. Among other results, we find that (i) the relative frequency of additive HGT increases with increasing phylogenetic distance, (ii) replacing HGT dominates at shorter phylogenetic distances, (iii) additive and replacing HGTs have strikingly different functional profiles, (iv) homologous recombination in flanking regions of a novel gene may be a frequent integration mechanism for additive HGT, and (v) phages and mobile genetic elements likely play an important role in facilitating additive HGT.

**Significance**

While many computational methods exist for large-scale inference of horizontal gene transfers (HGTs) between microbes, these methods do not currently classify the inferred HGTs as being additive or replacing. In this work, we address this gap by providing a new computational method that can rapidly classify a large fraction of HGTs as being additive or replacing with high confidence. We also leverage the new method to classify thousands of HGTs in *Aeromonas* and shed light on the relative frequencies, integration mechanisms, and functional biases of additive and replacing HGTs. By enabling quick, high-throughput classification of HGTs, this work will help further our understanding of HGT integration mechanisms and make it easier to interpret the functional impact of horizontally acquired genes in downstream studies.

## Introduction

*Horizontal gene transfer (HGT)* refers to the transfer of genetic material between two organisms that do not have a parent–offspring relationship. HGT can occur both intra- and inter-species, and is well-understood to be a major driver of microbial evolution. Transferred genes may be selfish genetic elements, or be associated with selfish genetic elements that facilitate integration into the recipient genome, and/or they may integrate into the recipient genome through homologous recombination. At a fundamental level, one can distinguish between *replacing* HGTs, where the transferred gene replaces an existing homologous gene in the recipient genome, and *additive* HGTs, where the transferred gene is added as a new gene to the recipient genome, possibly displacing one or more existing nonhomologous genes (Choi et al. 2012). However, classification of HGTs as being additive or replacing is nontrivial and there are currently no reliable, high-throughput computational solutions for this task. We note that the distinction between additive and replacing transfers does not necessarily correspond to their integration mechanisms as additive transfers can occur through homologous recombination in the flanking regions of the added gene (Polz et al. 2013). We also note that an additive transfer need not necessarily increase the number of genes in the recipient genome since the transferred gene may overwrite one or more nonhomologous genes, resulting in their immediate loss (Ely 2020).

Choi et al. (2012) studied the role of additive and replacing HGTs in the evolution of *Streptococcus*. That study was among the first to distinguish between the two types of HGTs, and adapted existing methods based on phylogenetic reconciliation and whole-genome alignments to do so (Didelot et al. 2010; Doyon et al. 2010). A key finding of the study was that replacing and additive HGTs appear to have played markedly different roles in the evolution of *Streptococcus* (Choi et al. 2012), with putative virulence genes being correlated with additive, not replacing, HGTs. Khayi et al. (2015) used phylogenetic discordance and gene order information to study additive and replacing HGTs in the pathogen *Dickeya solani* and suggested that inter-species HGT may have caused substantial variability in *Dickeya solani* genomes. More recently, there have been attempts at designing heuristics for distinguishing between additive and replacing HGTs using phylogenetic reconciliation (Kordi et al. 2019; Mondal et al. 2020), but such approaches do not make use of gene order information and currently have a high error rate even on simulated data. Thus, there do not yet exist any systematic, broadly applicable, rigorously tested approaches that can be used to reliably classify HGTs as additive or replacing. As a result, existing research on HGTs does not usually distinguish between additive and replacing HGTs, leaving their relative frequencies and specific roles in microbial evolution poorly understood.

In this work, we develop a novel computational framework that can classify a large fraction of HGTs inferred between the terminal edges of the phylogeny as being either additive or replacing with high confidence. Our framework leverages recent advances in HGT detection methods to identify high-confidence “terminal-edge” HGTs (i.e. HGTs occurring between branches leading to terminal taxa) and uses gene order information and phylogenetic relationships to classify a large fraction of the HGTs with high confidence. The resulting method, called *DART* (short for “Detection of Additive and Replacing Transfers”), is highly customizable, statistically supported, and scalable to hundreds of genomes and thousands of gene families.

We applied DART to a large dataset of over 7,500 gene families (homologous groups) from 103 *Aeromonas* strains representing 28 different species (Rangel et al. 2019; Kloub et al. 2021) and were able to classify a large fraction of the inferred terminal-edge HGTs with high confidence. Members of *Aeromonas* are found in water and sediments, live in symbiosis with fish, insects and leeches, and cause disease in humans and other animals (Janda and Abbott 2010; Milligan-Myhre et al. 2011; Marden et al. 2016; Fernandez-Bravo and Figueras 2020). The comprehensive *Aeromonas* dataset used in the current study (Kloub et al. 2021) has sufficient breadth and depth to assess both inter-species and intra-species HGTs. Moreover, HGT and recombination between members of *Aeromonas* occur frequently, making this an excellent test case (Morandi et al. 2005; Silver et al. 2011; Colston et al. 2014; Kloub et al. 2021).

Initial analysis of this dataset yielded a total of 31,187 high-confidence terminal-edge intra-species HGTs and 9,334 high-confidence terminal-edge inter-species HGTs. Among these, DART was able to classify 89% of the intra-species HGTs and 75% of the inter-species HGTs. Analysis of these classified HGTs provides insights into the relative frequencies, functional biases, and integration mechanisms of additive and replacing HGTs. Among other results, we find that (i) the relative frequency of additive HGT increases with increasing phylogenetic distance, (ii) replacing HGT dominates at shorter phylogenetic distances (e.g. between strains belonging to the same species), (iii) additive and replacing HGTs have strikingly different functional profiles, (iv) additive HGTs often, but not always, integrate themselves within gene neighborhoods similar to the original gene neighborhood in the donor genome, suggesting homologous recombination in flanking regions as a possible insertion mechanism, and (v) phages and mobile genetic elements likely play an important role in facilitating additive HGT, especially at larger phylogenetic distances. We also analyze two specific HGTs in detail and demonstrate how HGTs can sometimes have complex characteristics, simultaneously having additive, homologous replacement, and nonhomologous displacement components.

Overall, the ability to easily classify HGTs as either additive or replacing, as enabled by DART, has many potential benefits for microbial evolutionary studies. For example, it can provide insight into possible functions of transferred genes, improve our understanding of the evolutionary and functional implications of HGT (e.g. how often and under which conditions does HGT enable the acquisition of new abilities or functions versus refining existing abilities or functions?), and help refine our understanding of HGT integration mechanisms, particularly for additive HGTs. DART is open-source and freely available from https://compbio.engr.uconn.edu/software/dart/. The *Aeromonas* dataset and a complete list of classified HGTs are also freely available from the same URL.

## New Approaches

### Key ideas behind DART

Our classification framework makes use of (i) the absence and presence of homologs of the transferred gene in close phylogenetic neighbors (on the “species” or reference tree) of the recipient genome and (ii) the genomic context (i.e. gene neighborhood) of the transferred gene in the donor genome, recipient genome, and in the genomes of close phylogenetic neighbors of the recipient. Specifically, our classification of an HGT as replacing or additive is based on the following two simple observations, both of which follow logically from an understanding of microbial evolution and HGT. We point out that these observations only hold when a single gene (or small number of genes) is transferred by the underlying transfer event.

Observation 1 If homologs of the transferred gene are also present in some close phylogenetic neighbors of the recipient species and, furthermore, these homologs appear in similar gene neighborhoods in the recipient and its neighbors, then that transferred gene is likely to have been acquired as a replacing HGT.

Observation 2 If homologs of the transferred gene are not present in any of the most closely related phylogenetic neighbors of the recipient species then the transferred gene is likely to have been acquired as an additive HGT. Likewise, if homologs are present in one or more of the closest phylogenetic neighbors but none of those homologs appear in a similar gene neighborhood as in the recipient species, then the transferred gene is likely to have been acquired as an additive HGT.

As our results demonstrate, these two observations allow for the classification of a large fraction of terminal-edge HGTs, even when using conservative settings for homolog absence/presence and gene neighborhood conservation. We note that ideas similar to those of Observation 2 have also been previously used to show that, in bacteria and archaea, autochthonous gene duplications are rare relative to gene family expansion through additive HGT (Treangen and Rocha 2011; Tria and Martin 2021).

#### Exclusion of horizontal multigene transfers

It has previously been observed that many transferred genes are transferred as part of larger fragments containing several genes (Szöllősi et al. 2015; Brinkmann et al. 2018; Dunning et al. 2019; Kloub et al. 2021). Since accurately delineating the boundaries of such horizontal multigene transfers (HMGTs) remains a challenging problem, making it difficult to determine if conserved gene neighborhood is a result of undetected HMGTs, we focus on classifying only putative single-gene HGTs (but also including HGTs that are part of short HMGTs that are unlikely to affect classification accuracy). Accordingly, we first identify and filter out plausible HMGTs and only classify the remaining HGTs. We note that in many instances the gene is not the unit of transfer. If a transferred gene integrates into the recipient genome through homologous recombination, the recombination points are likely located inside the more conserved coding sequences and not in the less conserved intergenic regions (Gogarten et al. 2002).

#### Limiting classification to terminal-edge HGTs

DART takes into consideration the similarity of gene neighborhoods when classifying HGTs as additive or replacing. Furthermore, terminal-edge HGTs can be inferred with greater accuracy than nonterminal-edge HGTs, where there is often considerable ambiguity in exactly determining the donor and recipient “edges”, or lineages, on the species tree. We therefore focus our classification on only terminal-edge HGTs for which donors, recipients, and genome ordering information are unambiguously available. We discuss possible extensions of our framework to nonterminal-edge HGTs in the “Discussion” section.

### Key computational steps

**Fig. 1.**
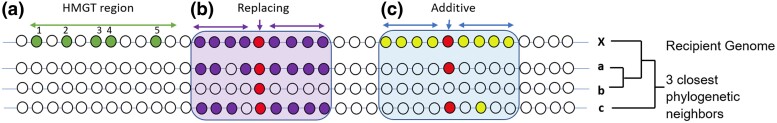
Overview of classification pipeline. The classification pipeline takes as input a list of terminal-edge HGTs for the genomes under consideration, gene order information for each genome, and phylogenetic relationships between the genomes. The figure depicts three different genomic regions from a hypothetical recipient genome *X*, along with corresponding genomic regions in the three closest phylogenetic neighbors of *X*. a) HGTs from some specific donor genome *D* (not depicted) are marked in green (shaded). Since these genes (green/shaded) acquired by *X* from *D* appear close together on the recipient genome, they may have been transferred as part of a single HMGT event. Such HGTs are therefore filtered out and not considered for classification. b) The transferred gene is shown in red on the recipient genome *X* and its eight immediate gene neighbors are shown in purple. Homologs of these genes are shown using the same colors (red and purple) in the three closest phylogenetic neighbors. As shown, in at least one (two in this example) of these three phylogenetic neighbors, the homolog of the transferred gene appears in the same genomic context (i.e. most of its nearest eight gene neighbors are purple). This HGT would therefore be classified as replacing. c) The transferred gene is shown in red on the recipient genome *X* and its eight immediate gene neighbors are shown in yellow. Homologs of these genes are shown using the same colors (red and yellow) in the three closest phylogenetic neighbors. As shown, for each of the three phylogenetic neighbors, either a homolog of the transferred gene does not exist in that genome or, if it does exists, occurs in a different genomic context (i.e. most of its closest eight gene neighbors are not yellow). This HGT would therefore be classified as additive. Note that this classification approach does not consider the donor genome.

We briefly describe the key steps in DART below:

1. *Inference of high-confidence terminal-edge HGTs.* We leverage recently developed reconciliation-based techniques, as implemented in the RANGER-DTL 2.0 software package (Bansal et al. 2018), to identify terminal-edge HGT events. RANGER-DTL 2.0 implements the DTL reconciliation framework that reconciles gene trees with a given species tree by invoking gene duplication, gene loss, and horizontal gene transfer events. Notably, DTL reconciliation has been shown to be highly effective in detecting (but not distinguishing between) both additive and replacing HGTs (Kordi et al. 2019). To filter out weakly supported HGTs, we account for several sources of HGT inference uncertainty including gene tree error, ambiguity of HGT detection, and uncertainty of assigning the donor and recipient for an HGT. Specifically, we error-correct all gene trees using TreeFix-DTL (Bansal et al. 2015), use a higher cost than the default for invoking HGT events in RANGER-DTL 2.0, and filter out all inferred HGTs that have less than 100% support.
2. *Mapping inferred HGTs to genomic locations.* For each inferred HGT, we identify the exact location of the transferred gene in the genomes (or gene orderings) of both the donor and the recipient (both of which are terminal taxa on the species tree). The small number of inferred HGTs for which exact genomic locations could not be unambiguously identified were discarded.
3. *Identifying and filtering out potential HMGTs.* An *HMGT* occurs when multiple complete genes are transferred in a single horizontal transfer event. We filter out those inferred HGTs that show signs of having been transferred from the same donor as part of an HMGT. This is done primarily based on the presence of additional plausible HGTs in the close vicinity of the transferred gene along the recipient genome. After this filtering step, we are left with a collection of high-confidence *classifiable* terminal-edge HGTs. Note that the HMGT filtering step is not perfect and the resulting set of “classifiable” HGTs consists not only of single-gene HGTs but likely also includes HGTs that are part of small undetected HMGTs. Such small undetected HMGTs are unlikely to affect classification accuracy.
4. *Assessing phylogenetic and genic neighborhood conservation.* For each inferred high-confidence classifiable terminal-edge HGTs, we quantify its *phylogenetic neighborhood conservation (PNC)* and *gene neighborhood conservation (GNC)*. To compute the PNC, we first identify the *m* closest phylogenetic neighbors of the recipient species/strain on the species tree and then determine how many of these *m* phylogenetic neighbors have at least one homolog of the transferred gene. We chose a default value of m=3 in our analysis. We compute GNCs between the recipient genome and each of its *m* phylogenetic neighbors that has a homolog, as well as between the recipient and donor genomes. Specifically, given a recipient genome *R* and another genome *G* that contains a homolog of the transferred gene, the GNC between *R* and *G* is computed by considering the set of the *n* closest neighboring genes of the transferred gene on the recipient genome and assessing how many of those neighboring genes are also neighbors of the homolog of the transferred gene in genome *G*. If *G* contains multiple homologs of the transferred gene then the maximum GNC across all homologs is used. We used a default value of n=8 (four genes on either side of the transferred gene) in our analysis.
5. *Classification of HGTs as additive, replacing, or ambiguous.* We use the PNC and GNCs computed for each high-confidence classifiable terminal-edge HGT to classify it as either additive, replacing, or ambiguous, based on appropriate (and customizable) classification thresholds. This classification approach is illustrated in Fig. 1. Specifically, in accordance with Observation 1, any HGT for which the PNC exceeds a certain threshold and at least one of the GNCs against the closest phylogenetic neighbors exceeds a certain threshold then that HGT is classified as a replacing HGT. In our analysis, by default, we required at least one of the three closest phylogenetic neighbors to have a homolog of the transferred gene, and at least one of the GNCs against the phylogenetic neighbors to be at least seven (i.e. at least seven of the eight closest neighboring genes of the transferred gene must also be among the eight closest gene neighbors of at least one of the homologs in the closest phylogenetic neighbors). Likewise, in accordance with Observation 2, any HGT for which either the PNC is below a certain low threshold or all GNCs against phylogenetic neighbors with homologs are below a certain threshold is classified as an additive HGT. In our analysis, by default, we set the PNC threshold to 0 (i.e. no homologs found in any of the three closest phylogenetic neighbors) and the GNC threshold to be at most 1. All HGTs that cannot be classified as additive or replacing using the chosen thresholds are classified as *ambiguous*.
6. *Statistical analysis and post-processing.* We perform statistical analysis through randomization of the inferred HGTs to estimate the false-positive classification rate and determine/justify the thresholds used for the classification so as to achieve desired trade-offs between false-positive and false-negative classifications. Furthermore, to account for potential classification errors due to data quality issues (such as incomplete gene calling or under-clustering of gene families), we show how to perform additional filtering of DART’s classification results using BLAST, gene orderings for donor genomes, and other information.

Further details appear in the Materials and Methods section.

## Results

We applied DART to a large dataset of 7,567 consolidated homologous groups (cHGs), i.e. gene families, each containing at least 4 genes, from 103 *Aeromonas* genomes (Kloub et al. 2021). The 103 genomes in this dataset represent 28 different species. Among these 28 species, 18 are represented by a single genome and the remaining 10 are represented by at least two genomes each from different strains belonging to that species. This allows us to distinguish between HGTs that are *intra-species*, i.e. where the donor and recipient genomes are from the same species, and those that are *inter-species*, i.e. where the donor and recipient genomes are from different species.

As described in the Materials and Methods section, we identified a total of 40,521 high-confidence classifiable terminal-edge HGTs with known gene IDs among the 7,567 cHGs in our dataset. Among these 40,521 HGTs, 9,334 are inter-species HGTs and the remaining 31,187 are intra-species HGTs. As described in detail below, we found that DART is able to classify a large fraction of these inter- and intra-species HGTs even with our conservative default parameter settings.

### DART can Classify the Vast Majority of Terminal-Edge HGTs

**Fig. 2.**
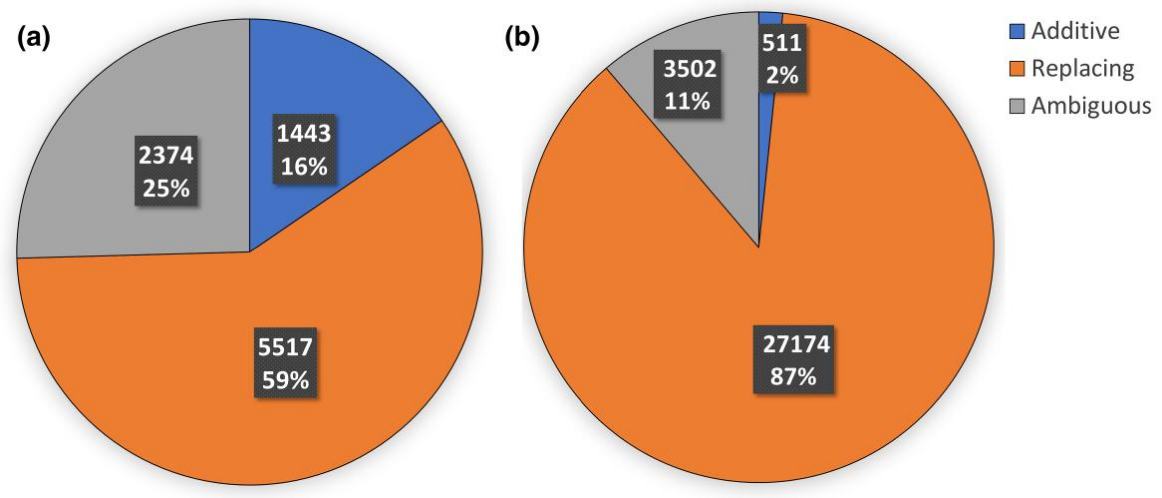
Classification results for inter- and intra-species HGTs. The pie charts show the fractions of all inter-species a) and intra-species b) HGTs classified as additive, replacing, and ambiguous using DART with default parameter settings. The first part of each slice label is the total number of HGTs in the corresponding category and the second part is the percent area of the pie occupied by that slice. a) Inter-species HGTs and b) Intra-species HGTs.

Using our default parameter settings (as described in detail in the Materials and Methods section), DART was able to classify 6,960 (74.6%) of the 9,334 inter-species HGTs and 27,685 (88.8%) of the 31,187 intra-species HGTs as either additive or replacing (Fig. 2). Thus, DART is able to classify the vast majority of the HGTs, even with our use of conservative default parameter settings.

As Fig. 2 shows, we find that intra-species HGTs are predominantly classified as being replacing, with 27,174 (98.1%) of the 27,685 classified intra-species HGTs being classified as replacing compared to only 511 (1.85%) classified as additive. In contrast, a much larger fraction of inter-species HGTs is classified as additive, with 1,443 (20.7%) of the 6,960 classified inter-species HGTs classified as additive and 5,517 (79.3%) classified as replacing. These results are consistent with the expectation that closely related strains belonging to the same species would engage in HGT primarily through replacing homologous recombination, while more distantly related strains with more divergent gene contents would be less able to engage in replacing homologous recombination and consequently show a higher rate of additive HGT. Later in this section, we more systematically assess the relationship between phylogenetic distance (or divergence) between donor and recipient and the relative frequencies of additive and replacing HGTs.

### Classification Results are Robust to Parameter Settings

DART uses four key parameters to classify HGTs as additive or replacing (or ambiguous): (i) number of phylogenetic neighbors, *m*, of the recipient to use to compute PNC, (ii) gene neighborhood size, *n*, to assess GNC, (iii) classification threshold for additive HGTs, and (iv) classification threshold for replacing HGTs. These parameters are described in detail in the Materials and Methods section. To assess the robustness of the above classification results to changes in these parameter settings, we considered three different settings each for *m*, specifically m=1,2, and 3 (default), three different settings for *n*, specifically n=4,8 (default), and 16, and four different settings for the classification thresholds, specifically (additive, replacing) =(0%,100%),(<10%,>90%),(<20%,>80%) (default), and (<30%,>70%). Table 1 and supplementary Table S2, Supplementary Material online show the results of this analysis for inter-species and intra-species HGTs, respectively.

**Table 1:** DART classification results for inter-species HGTs using different parameter settings

	Additive threshold = 0%, Replacing threshold = 100%		
	m=1	m=2	m=3
n=4	2,353, 4,250, 2,731	1,953, 5,022, 2,359	1,689, 5,396, 2,249
n=8	1,961, 2,684, 4,689	1,639, 3,446, 4,249	1,403, 3,818, 4,113
n=16	1,666, 1,098, 6,570	1,437, 1,570, 6,327	1,238, 1,853, 6,243

	Additive threshold < 10%, Replacing threshold > 90%		
	m=1	m=2	m=3
n=4	2,353, 4,250, 2,731	1,953, 5,022, 2,359	1,689, 5,396, 2,249
n=8	1,961, 2,684, 4,689	1,639, 3,446, 4,249	1,403, 3,818, 4,113
n=16	1,679, 2,335, 5,320	1,454, 3,077, 4,803	1,260, 3,420, 4,654

	Additive threshold < 20%, Replacing threshold > 80%		
	m=1	m=2	m=3
n=4	2,353, 4,250, 2,731	1,953, 5,022, 2,359	1,689, 5,396, 2,249
n=8	1,990, 4,437, 2,907	16,69, 5,211, 2,454	**1,443, 5,517, 2,374**
** n=16 **	1,714, 3,711, 3,909	1,494, 4,503, 3,337	1,308, 4,793, 3,233

	Additive threshold < 30%, Replacing threshold > 70%		
	m=1	m=2	m=3
n=4	2,533, 5,691, 1,110	2,104, 6,298, 932	1,853, 6,556, 925
n=8	2,059, 5,249, 2,026	1,736, 5,918, 1,680	1,515, 6,184, 1,635
n=16	1,731, 4,140, 3,463	1,515, 4,895, 2,924	1,336, 5,195, 2,803

Results are shown for 36 different parameter setting combinations for number of phylogenetic neighbors *m*, gene neighborhood size *n*, additive HGT classification threshold, and replacing HGT classification threshold. Each cell shows three comma-separated values representing the number of additive, replacing, and ambiguous HGTs, respectively. The cell with numbers in bold font represents default parameter settings.

As Table 1 shows, neither parameter *m* nor parameter *n*, when changed individually from their default values, has a drastic impact on inter-species HGT classification results. This shows the robustness of the classification results produced by DART. Intra-species classification results, shown in supplementary Table S2, Supplementary Material online, also support classification robustness, remaining largely unaffected by parameter *n* though they are more greatly affected by parameter *m*. As expected, the number of HGTs that can be classified as either additive or replacing increases as the additive and replacing classification thresholds are made less restrictive. For instance, for default values of the other parameters, the number of inter-species additive and replacing HGTs increases slightly from 1,443 and 5,517, respectively, using default threshold parameters to 1,515 and 6,184, respectively, when using the more permissive thresholds of <30% for additive HGTs and >70% for replacing HGTs. Likewise, for intra-species HGTs, the corresponding numbers increase slightly from 511 and 27,174 to 520 and 28,403, respectively.

Among all 36 combinations of the tested parameter settings, the most permissive for additive classification is when m=1, n=4, and additive classification threshold is <30%, and the most restrictive is when m=3, n=16, and additive classification threshold is 0%. These settings result in 2,533 and 1,238 inter-species HGTs being classified as additive, respectively, as compared to 1,443 for default parameter values. For intra-species HGTs these numbers are 1,698 and 484, respectively, compared to 511 for default parameter values. For replacing HGTs, the most permissive classification is when m=3, n=4, and replacing classification threshold is >70%, and the most restrictive is when m=1, n=16, and additive classification threshold is 100%. These settings result in 6,556 and 1,098 inter-species HGTs being classified as replacing, respectively, as compared to 5,517 for default parameter values. For intra-species HGTs, these numbers are 29,288 and 9,618, respectively, compared to 27,174.

### Estimating False-Positive Rate Using Statistical Analysis

We performed statistical analysis to further validate our choice of default parameter values and overall classification approach. Specifically, we used randomization to estimate the false-positive rate for the additive and replacing HGT classifications. Such false-positive classifications can occur, for instance, when a gene is acquired additively but one of the phylogenetic neighbors of the recipient also has a homolog of that gene in a similar gene neighborhood, or when a gene is acquired as replacing but none of the homologs in the phylogenetic neighbors of the recipient appear in a similar gene neighborhood.

As described in detail in the Materials and Methods section, our randomization analysis for estimating the false-positive rate for replacing HGTs randomizes the 40,521 HGTs considered in our analysis, preserving total counts as well as donors and recipients, so that the randomized HGTs mimic characteristics of additive HGTs, and checks how often such “additive HGTs” get classified as replacing using DART. Analogously, our randomization analysis for estimating the false-positive rate for additive HGTs randomizes the 40,521 HGTs, preserving total counts as well as donor and recipients, so that the randomized HGTs mimic characteristics of replacing HGTs, and checks how often such “replacing HGTs” get classified as additive using DART. We performed this randomization analysis 100 times each for additive and replacing HGTs and averaged over the resulting rates.

Using default parameter settings for DART, our randomization analysis resulted in false-positive rates of 0.84% and 0.44% for inter-species and intra-species additive HGTs, respectively, and only 0.04% and 0.06% for inter-species and intra-species replacing HGTs, respectively. These results show that the classifications resulting from DART, when applied to this *Aeromonas* dataset using default parameters, are highly robust to incorrect classification due to chance matches or mismatches of gene neighborhoods. Complete results for all 36 combinations of tested parameter settings appear in supplementary Tables S3, Supplementary Material online, S4, S5, and S6. These complete results show that additive and replacing classifications computed by DART on the *Aeromonas* dataset remain remarkably robust even for permissive parameter values, with estimated false-positive rates never exceeding 4.52% for additive HGTs and 0.21% for replacing HGTs. Note, however, that the number of HGTs classified as ambiguous varies widely for different parameter settings for the additive HGT analysis, both inter- and intra-species.

We point out that the reported false-positive rates are dataset specific and can vary across different datasets depending on gene content conservation and gene ordering conservation of the genomes under consideration.

### Post-processing and Additional Filtering of Classified HGTs

The classification approach used by DART relies on correctly estimating PNC and GNC for the HGT being classified. Incomplete gene calling or potential under-clustering of gene families can lead to erroneous assessments of PNC and GNC, leading to incorrect HGT classification. This is particularly true for HGTs classified as additive since incomplete gene calling or potential under-clustering of gene families can artificially decrease PNC and/or GNC. Likewise, contig breaks close to homologs of the transferred gene in the phylogenetic neighbors of the recipient or undetected HMGTs can also affect PNC and/or GNC calculations.

To filter out potential cases of classification error we performed post-processing of the classified HGTs and filtered out HGTs that showed any indication of having been misclassified. We describe the specific steps of our post-processing pipeline and the results of this analysis below.

**Post-processing Step 1:**  *Finding missing homologs using BLAST.* To account for incomplete gene calling or under-clustered gene families, we used BLAST (Altschul et al. 1990) to verify the absence of any relevant homologs of the transferred gene in the genomes of interest. Specifically, we used BlastN to identify possible undetected homologs of the transferred gene in all *m* phylogenetic neighbors of the recipient and then evaluated their gene neighborhoods. Any hit that had an E-value of less than $1e^{-10}$ and an overlap of at least 70% with the aligned query sequence (transferred gene) was deemed a possible homolog of the transferred gene.
Observe that undetected homology does not affect HGTs classified by DART as replacing. Thus, we used this approach to identify those HGTs that may be have been erroneously classified as additive due to possible undetected homology. For any new homolog discovered using BLAST, we further used BLAST to identify possible undetected homologs of the *n* neighboring genes of the transferred gene along the recipient genome within 10,000 bp on either side of the newly discovered homolog. If any of the new homologs also had some conserved neighboring genes (more than additive classification threshold for GNC), as discovered using BLAST, then that HGT was labeled as ambiguous (i.e. unclassified). Among the 1,443 inter-species additive HGTs inferred by DART, we found that 296 had possible undetected homologs with at least some gene neighborhood conservation in one or more of the 3 closest phylogenetic neighbors of the recipient genome. Among the 511 intra-species additive HGTs inferred by DART, 173 such cases were found. These were all filtered out of the list of additive HGTs and marked as ambiguous. It is important to note that this BLAST-based filtering criteria is quite strict since we expect many of the new homologs to represent distant ancient paralogs or other similar sequences. Thus, many of the additive HGTs that are filtered out were likely correctly classified as additive.
**Post-processing Step 2:**  *Fine grained analysis of homologs close to contig ends.* DART takes proximity to contig ends into consideration when classifying HGTs. In some cases, this could lead to situations where the PNC and GNC calculations are based on only a single closest phylogenetic neighbor of the recipient neighbor, because the other phylogenetic neighbors under consideration had contig ends too close to homologs of the transferred gene. In such cases, the HGT may be classified as additive based on the single phylogenetic neighbor (note that replacing HGT classifications are unaffected by this issue). We therefore, checked to see if the remaining phylogenetic neighbors that were removed from consideration in PNC and GNC calculations due to proximity of contig ends did, in fact, support the classification of the transferred gene as additive. Specifically, we checked gene neighborhood conservation for these contig-end cases and found that 21 of the inter-species HGTs classified as additive and 21 of the intra-species HGTs classified as additive had at least one phylogenetic neighbor that exceeded the additive classification threshold for GNC. These 21 inter-species and 21 intra-species cases were therefore filtered out and marked as ambiguous.
**Post-processing Step 3:**  *Identifying potential undetected HMGTs.* DART can result in incorrect classifications if the HGTs being classified were transferred as part of large fragments, or large HMGTs, containing multiple genes. Despite our initial filtering of potential HMGTs, we found that some of the HGTs classified as additive appeared to be part of HMGTs in that several of the neighboring genes of the transferred gene were absent from the closest phylogenetic neighbors of the recipient genome. This indicates that those missing neighboring genes may have been transferred along with the identified HGT gene. To exclude such cases of potential HMGT from classification, we used BLAST (using the same settings as described above) to identify those HGTs that were classified as additive but none of whose three closest phylogenetic neighbors contained homologs of the two closest genes flanking the HGT gene along the recipient genome. Surprisingly, we found many such potential HMGT cases; 474 among inter-species additive HGTs and 132 among intra-species additive HGTs. We filtered out these potential HMGT cases from the list of additive HGTs. We note, however, that most of these potential HMGT cases are likely additive, i.e. their classification was likely correct.
**Post-processing Step 4:**  *Validating replacing HGTs using donor gene orderings.* Finally, we used the known gene ordering along the donor genome of each HGT classified as replacing to further validate if that HGT is classified correctly, In particular, in case of replacing HGTs, we expect the GNC between the donor genome and the recipient genome to be high (above the replacing HGT classification GNC threshold). Note that this need not necessarily hold true for all replacing HGTs, but we found that it did hold true for all but 28 of the 5,517 inter-species replacing HGTs and all but 2 of the 27,174 intra-species replacing HGTs. In keeping with the very strict filtering implemented in previous steps, we filtered out these 30 cases from the list of replacing HGTs.

**Fig. 3.**
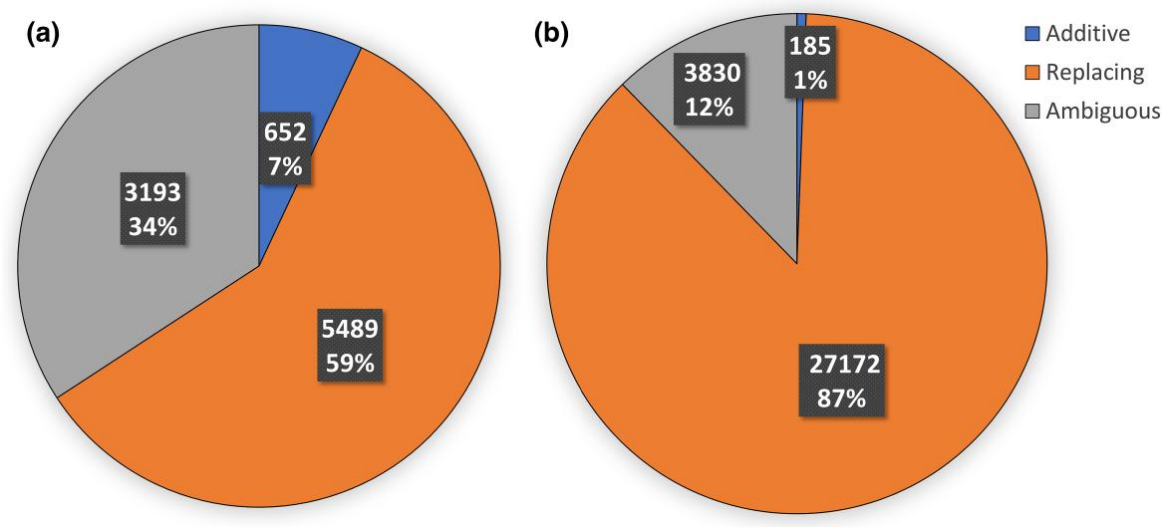
Filtered classification results for inter- and intra-species HGTs. The pie charts show the fractions of all inter-species a) and intra-species b) HGTs classified as additive, replacing, and ambiguous after post-processing and aggressively filtering DART’s initial classification results computed using default parameter settings. The first part of each slice label is the total number of HGTs in the corresponding category and the second part is the percent area of the pie occupied by that slice. a) Inter-species HGTs and b) Intra-species HGTs.

Thus, based on our very strict filtering of possible HMGTs or possible erroneous classifications, we filter out 791 of the 1,443 inter-species additive HGTs, 326 out of the 511 intra-species additive HGTs, 28 out of the 5,517 inter-species replacing HGTs, and 2 out of the 27,174 intra-species replacing HGTs as classified by DART. Figure 3 summarizes these filtered classification results. In addition, supplementary Figures S1 and S2, Supplementary Material online show how HGTs classified as additive are distributed among different intra-species and inter-species donor–recipient pairs, respectively. Corresponding figures are not provided for replacing HGTs due to the very large number of HGTs.

In the remainder of the Results section, we use this filtered list of additive and replacing HGTs for further analysis. Importantly, we also found that all of the subsequent observations hold even if the full, unfiltered list of additive and replacing HGTs is used for these analyses.

### Relative Frequency of Additive HGTs Increases with Phylogenetic Distance

We noticed that the filtered classification results were consistent with our initial observation (on the unfiltered classification results) that HGTs are additive with far greater relative frequency inter-species than intra-species (Fig. 3). In other words, a much greater proportion of inter-species HGTs are additive compared to intra-species HGTs. Before analyzing the Aeromonas dataset, we had hypothesized the following:

**Hypothesis:** The relative frequency of additive HGT increases with increasing phylogenetic distance between donor and recipient.

**Fig. 4.**
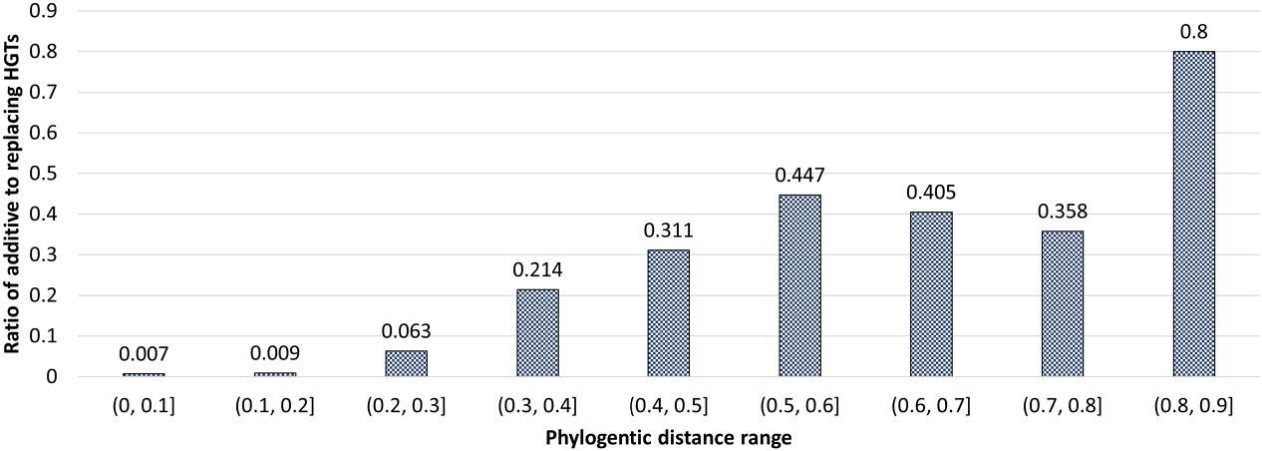
Fraction of additive HGTs by phylogenetic distance. The plot shows the fraction of HGTs classified as additive for donor–recipient pairs separated by different phylogenetic distance ranges. Results are shown for the combined set of filtered inter- and intra-species HGTs classified as additive and replacing. The phylogenetic distance between any donor–recipient pair is the patristic distance (with branch lengths representing substitutions per site) between the two corresponding terminal taxa on the species tree.

A fine grained analysis of the relative frequencies of additive and replacing HGTs at different phylogenetic distance ranges strongly supports this hypothesis. These results are shown in Fig. 4. As the figure shows, the fraction of HGTs that are classified as additive consistently increases with increasing phylogenetic distance, with additive HGTs constituting less than 1% of all classified HGTs for the smallest phylogenetic distance range but 80% of all classified HGTs for the largest phylogenetic distance range. This dramatic increase in the relative frequency of additive HGTs is driven by a rapid decline in the number of replacing HGTs with increasing phylogenetic distance. For instance, in the (0,0.1] phylogenetic distance range we see 118 additive and 16,995 replacing HGTs, but in the (0.2,0.3] distance range, we see 105 additive and only 1,561 replacing HGTs.

The corresponding plot for full, unfiltered classification results is shown in supplementary Figure S3, Supplementary Material online and shows that the hypothesis remains strongly supported even when using unfiltered classification results.

### Additive HGTs often, but not Always, Show Conserved Genomic Context

It is reasonable to expect that replacing HGTs should, overwhelmingly, insert themselves in a similar genomic context in the recipient genome as in the donor genome. Indeed, the results of applying post-processing Step 4 for additional filtering, as reported earlier, confirm this expectation. However, it is not generally known if additive HGTs also usually insert themselves within similar genomic contexts or how often they do not. We therefore used our filtered classification results to check how often additive and replacing HGTs have the same two flanking genes in their donor and recipient genomes. Table 2 shows the results of this analysis. As the results show, 99.5% of intra-species replacing HGTs and 98.1% of inter-species replacing HGTs had at least one flanking gene in common between donor and recipient, but only 88.6% of intra-species additive HGTs and 73.5% of inter-species additive HGTs had at least one gene in common. Thus, up to 26.5% of inter-species additive HGTs appear to have inserted in regions of low genomic context similarity. This discrepancy between replacing and additive HGTs holds even if we account for the phylogenetic distance between donor–recipient pairs (third and sixth rows of Table 2), and even if unfiltered classification results are used (supplementary Table S7, Supplementary Material online). Nonetheless, the overall high level of genomic context conservation even among additive HGTs supports the hypothesis that insertion of many additively acquired genes may occur via homologous recombination in flanking regions (Polz et al. 2013). We note, however, that undetected HMGTs can inflate the number of HGTs showing conserved genomic context and so these results should be interpreted with caution.

**Table 2:** Genomic context conservation results for filtered additive and replacing HGTs

	Both conserved (%)	At least one conserved (%)	No conservation (%)
Intra-species additive	65.9	88.6	11.4
Inter-species additive	37.1	73.5	26.5
Phylo. distance ≥0.5 additive	24.4	65.7	34.3
Intra-species replacing	89.3	99.5	0.5
Inter-species replacing	81.6	98.1	1.9
Phylo. distance ≥0.5 replacing	74.2	97	3

For each category (row) of additive and replacing HGTs, the table reports the percentage of HGTs that (i) have the same two flanking genes in both donor and recipient genomes, (ii) have at least one of the two flanking genes in common between donor and recipient, and (iii) have none of the two flanking genes in common between donor and recipient. “Phylo. distance” refers to the phylogenetic distance between donor and recipient genomes on the species tree and is defined to be the patristic distance (with branch lengths representing substitutions per site) between the two corresponding terminal taxa on the tree.

### Additive and Replacing HGTs have Functional Biases

**Fig. 5.**
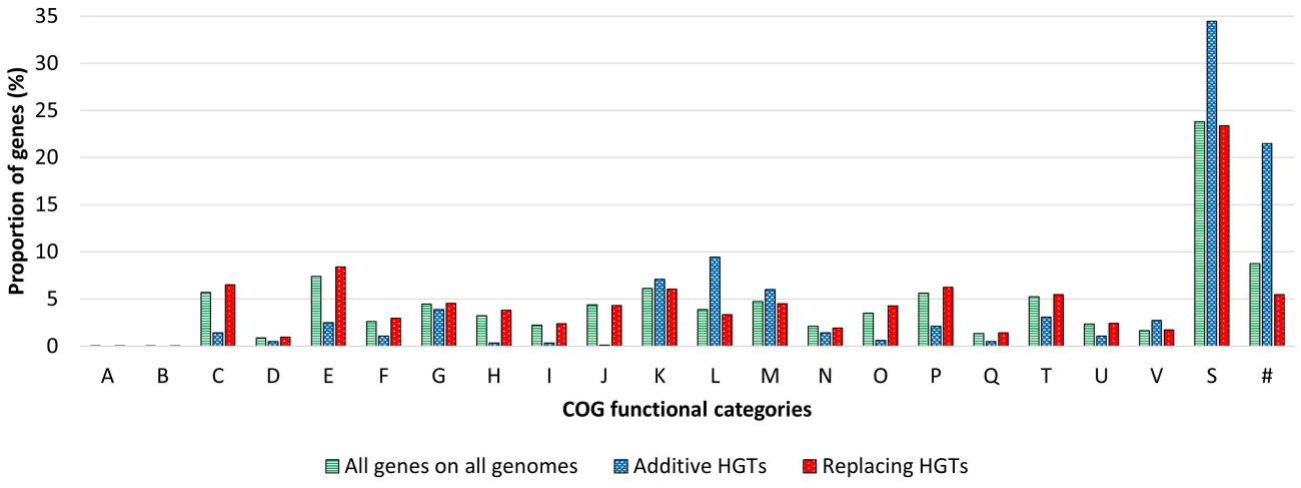
Functional analysis of additive and replacing HGTs. The figure shows distributions of COG functional categories for (i) all genes from all genomes, (ii) all HGTs classified as additive, (iii) all HGTs classified as replacing. Only HGTs present in the filtered classification results were used, and both intra- and inter-species HGTs are included. Each letter corresponds to a COG functional category as shown in supplementary Table S1, Supplementary Material online. Some key functional categories are also shown in Table 3. COG functional categories “Z”, “Y”, “W”, and “R” are not shown since no gene in any of the *Aeromonas* genomes belonged to those categories. COG Functional category “S” corresponds to genes whose functions are unknown, while the category “#” corresponds to genes which could not be assigned to any COG functional category.

It has previously been observed that additive and replacing HGTs appear to play different roles in *Streptococcus* evolution (Choi et al. 2012). In general, it is reasonable to expect that core genes would be more likely to be transferred as replacing while gene families with patchy distributions within a clade of interest would be acquired additively. However, there have been no large-scale, systematic studies of fine grained functional biases of additive and replacing HGTs. We therefore used the over 33,000 HGTs in our filtered classification results to study which COG functional categories were substantially over- or under-represented among additive and replacing HGTs. Figure 5 plots the distribution of COG functional categories in additive and replacing HGTs. For comparison, the figures also plot the distribution of COG functional categories for all genes on all genomes in the dataset. The figure shows that there are clear differences between the functional distributions of additive and replacing HGTs. For a more systematic analysis, we identified all those functional categories that were enriched by at least a factor of two among either additive or replacing HGTs; these are reported in Table 3. As Fig. 5 shows, a much larger fraction of additive HGTs could not be assigned a function (assigned instead to categories “S” or “#” in Fig. 5), thereby artificially lowering the proportion of genes in the other functional categories. For a fair functional comparison between additive and replacing HGTs, the enrichment factors reported in Table 3 are therefore based on the 24,174 additive and replacing HGTs that could be assigned to a COG category of known function (i.e. not assigned to categories “S” or “#” in Fig. 5). The corresponding plot without “S” and “#” categories appears in supplementary Figure S4, Supplementary Material online. A description of each COG functional category appears in supplementary Table S1, Supplementary Material online.

**Table 3:** Functional enrichment in additive and replacing HGTs

Enriched in replacing HGTs	
COG functional category	Enrichment factor
[J] Translation, ribosomal structure, and biogenesis	22.7×
[H] Coenzyme transport and metabolism	6.7×
[O] Post-translational modification, protein turnover, chaperone	4.5×
[I] Lipid transport and metabolism	4.2×
[C] Energy production and conversion	2.8×
[E] Amino acid transport and metabolism	2.1×
Enriched in additive HGTs	
[L] Replication, recombination, and repair	4.6×
[V] Defense mechanisms	2.5×
[M] Cell wall/membrane/envelope biogenesis	2.2×

The table reports all COG functional categories that are enriched by a factor of at least two in replacing HGTs (top) and additive HGTs (bottom). Reported enrichment factors are based on a total of 24,174 additive and replacing HGTs that could be assigned to a proper COG functional category (i.e. not assigned to categories “S” or “#” in Fig. 5). Functional categories “A” and “B” were not considered since they were not well represented among the additive and replacing HGTs (fewer than five genes).

As Table 3 shows, [J], [H], [O], [I], [C], and [E] functional categories are substantially enriched (at least 2×) in replacing HGTs compared to additive HGTs. Notably, functions [J], [H], [O], and [I] enriched at more than 4×. Among additive HGTs, we found the [L], [V], and [M] functional categories to be substantially enriched in additive HGTs compared to replacing HGTs, with function [L] enriched at more than 4× (Table 3). These findings are consistent with the expectation that HGTs involving core genes should most often be replacing since additive HGTs of such genes would alter the gene’s dosage and thus likely be deleterious to the recipient’s fitness. Nearly all genes in the [J] and [H] functional categories and most genes in the [C], [I], and [E] categories can be viewed as being core genes. Likewise, the result for additive HGTs is consistent with the expectation that selfish genetic elements (SGEs), which would fall under this [L] category, are likely to be acquired through additive HGT. The preponderance of additive transfers in the [M] and [V] categories likely reflects the ongoing arms race between cellular organisms and their defense systems on one side and viruses and SGEs on the other (Koonin et al. 2017). Defense systems that can inactivate viruses and SGEs often have a sporadic distribution within populations (Kong et al. 2013; Fullmer et al. 2019), preventing whole populations from being wiped out by a virus. While these systems provide some protection to the organisms that carry them, they also make them vulnerable to parasites that have adapted to the particular defense mechanism (an early observation of this learning by phages is in Bertani and Weigle (1953)). Similarly, changes in the cell wall and surface structure of cellular organisms may protect against a virus that uses this structure as a signal or for attachment. That the acquisition of components of the arms race in the cellular genomes is frequently additive reflects the vast diversity of mechanisms carried out by isofunctional but nonhomologous or only distantly related proteins.

In addition to the above, observe that a much larger fraction of additive HGTs, almost 4× compared to replacing HGTs, could not be assigned to any COG functional category (# functional category in the figures). This suggests that a much larger fraction of additively acquired genes do not have known functions. This observation is consistent with the above findings and may reflect the fact that a larger fraction of additive HGTs are noncore genes which are not as well characterized.

Note that the functionally enriched categories identified in Table 3 are unlikely to be seen by chance alone, especially those identified at more than 4× enrichment. This is due to the large number (24,174) of additive and replacing HGTs on which this analysis is based and due to our use of stringent enrichment thresholds (2× and 4×). Furthermore, we find that nearly identical functional enrichment patterns hold even when the analysis is repeated using only intra-species or only inter-species HGTs (supplementary Figures S5 and S6, and Table S8, Supplementary Material online).

### Qualitative Assessment of Additive and Replacing HGTs

To gain further insight into the nature of the different kinds of HGTs, we randomly sampled 200 individual HGT events (50 each of inter-species additive, inter-species replacing, intra-species additive, and intra-species replacing) and assessed them qualitatively. In addition, we used 50 randomly selected genes as control. We observed that both additive and replacing transfer frequently made use of mobile genetic elements and phages as vehicles of transfer but that the prevalence of phages and mobile genetic elements was much higher for inter-species additive HGTs than for inter-species replacing HGTs. Specifically, out of 50 randomly selected inter-species additive HGTs, 28 were confirmed to be or were adjacent to (within 20 KB, or less if contig end was nearer) phage integrases, prophage genes, recombinases, or restriction modification system genes. However, among the 50 inter-species replacing HGTs, only 6 were confirmed to be associated with those mechanisms. Instead, inter-species replacing HGTs were more often located near tRNAs (22 out of 50) compared to inter-species additive HGTs (14 out of 50). This suggests that some of the inter-species replacing HGTs may be the result of larger scale chromosome recombination events which tend to be associated with nearby tRNAs (Reiter et al. 1989; Williams 2002; Rodriguez-Valera et al. 2016). Among the control set of 50 randomly chosen genes, only 9 were associated with phages or mobile genetic elements, and 11 were associated with tRNAs.

Interestingly, we saw little qualitative differences between intra-species additive and intra-species replacing HGTs. Among the 50 intra-species additive HGTs, 12 were associated with phages or mobile genetic elements and 14 with tRNAs. Results were nearly identical for the 50 intra-species replacing HGTs, with 11 associated with phages or mobile genetic elements and 12 with tRNAs. This lack of bias between intra-species additive and replacing HGTs may reflect a lessened role of phages or mobile genetic elements for intra-species transfer.

### Displacing Additive HGTs: Two Case Studies

Recall that an additively transferred gene can add itself to the recipient genome either without affecting any existing genes or by displacing one or more nonhomologous genes (Ely 2020). We refer to the latter kind of additive HGT as a *displacing additive HGT*. While many of the HGTs classified as additive show clear evidence of not being displacing (results not shown), we found several convincing examples of additive HGTs that appear to have displaced a nonhomologous gene. Specifically, we observed that for some of the additive HGTs (using filtered classification results), at least two of the three closest phylogenetic neighbors of the recipient had a gene from a different cHG (i.e. gene family) present in the “same” genomic position as the transferred gene (i.e. in genomic positions that exceeded the additive classification threshold for GNC). Using this conservative criterion, we found that 11 of the filtered inter-species additive HGTs and 11 of the filtered intra-species additive HGTs could be inferred to be displacing additive HGTs. We investigated two such displacing additive HGTs, one inter-species and one intra-species, in detail and discuss our findings below. In both cases, we find that these additive HGTs likely resulted in both homologous replacement and nonhomologous displacement. These case studies highlight some of our hypothesized mechanisms and illustrate how some HGTs may require more nuanced classification.

*HGT in cHG 18,292 from Aeromonas veronii F247 to Aeromonas veronii CIP107763.* Alignment dot plots of the donor, recipient, and its three closest phylogenetic neighbors (supplementary Figure S7, Supplementary Material online) show regions of duplicated DNA sequences near the site of the insertion. These regions might result in hairpin loop structures of the DNA which would help to facilitate a homologous recombination event. A closer analysis using our gene annotations (both RAST Aziz et al. 2008 and a follow up using Prokka Seemann 2014) reveals a somewhat complex transfer event (supplementary Figures S8 and S9, Supplementary Material online). The region around a AAA ATPase, along with 2 hypothetical proteins, appears to have been replaced in the recipient by a divergent AAA ATPase homolog and a new hypothetical gene from the donor sequence. Thus, this additive HGT appears to have homologous replacement and nonhomologous displacement components (supplementary Figure S9, Supplementary Material online).

*HGT in cHG 21,480 from Aeromonas taiwanensis LMG24683T to Aeromonas enteropelogenes CECT4487T.* Dot plots of the donor, recipient, and neighboring genomes are shown in supplementary Figure S10, Supplementary Material online. Initially, it appears that a single gene (either a dihydrofolate reductase, or riboflavin biosynthesis protein depending on the annotation software used) was inserted in-between an alternative ribosomal rescue factor and a HTH-type transcriptional regulator (supplementary Figure S11, Supplementary Material online). However, closer inspection of these genes and their annotations (supplementary Figure S12, Supplementary Material online), indicates that the transfer is not that simple. BLAST (Altschul et al. 1990) searches of the gene neighborhood around and including the transferred gene indicate that the recipient’s copy of the alternative ribosomal rescue factor is much more closely related to the donor than any of the neighboring genomes, including the other member of its species. Hence, it appears that an additive HGT brought in cHG 21,480, while also replacing all or part of the alternative ribosomal rescue factor from *A. enteropelogenes* with a copy from *A. taiwanensis*. Thus, this additive HGT has a homologous replacement component. Interestingly, our analysis erroneously flags this HGT as being displacing additive since homologs of the alternative ribosomal rescue factor in the three phylogenetic neighbors of the recipient appear in a different cHG than the alternative ribosomal rescue factor homolog in the recipient. Thus, the flagging of this HGT as a displacing additive HGT is a result of over-splitting of the alternative ribosomal rescue factor gene family in our dataset. This case study also highlights how the use of different annotation software can result in different gene calls and functional inferences (supplementary Figure S12, Supplementary Material online).

Methodological details related to the Prokka annotations and Gepard plots used for the above case studies (supplementary Figures S8, S9, S11, and S12, Supplementary Material online) appear in the Supplementary Material online.

## Discussion

In this work, we introduced a new, high-throughput approach, called DART, for classifying inferred HGTs as additive or replacing. Our analysis of the *Aeromonas* data shows that DART can confidently classify a large fraction of terminal-edge HGTs as either additive or replacing, and reveals several important insights into the prevalence, functional characteristics, and integration mechanisms of additive and replacing HGTs. For instance, we find that the fraction of HGTs acquired additively increases with increasing phylogenetic distance, and that replacing HGTs greatly dominate among strains or genomes from the same or closely related species. We also find clear functional preferences among replacing HGTs and additive HGTs, with enrichment in one or the other across several different COG categories. Interestingly, we find that a much larger fraction of additively acquired genes have poorly characterized or unknown functions. Our analysis also suggests that, not necessarily surprisingly, even additively acquired genes often integrate themselves within gene neighborhoods that are similar to their gene neighborhoods in donor genomes, pointing to homologous recombination in flanking regions as a possible insertion mechanism. We also find that phages and mobile genetic elements likely play an important role in facilitating additive HGTs, especially at larger phylogenetic distances. Moreover, our case studies highlight how some HGTs can have complex characteristics, simultaneously having additive, homologous replacement, and nonhomologous displacement components (supplementary Figures S7 through S11, Supplementary Material online). The resulting software is easy to use and highly customizable and simplifies the inference of additive and replacing HGTs; we expect that its application will lead to improved understanding of the functional potential, integration mechanisms, and evolutionary impacts of HGT. In particular, analysis of additional microbial datasets from other genera could further refine the insights from our *Aeromonas* analysis and help identify general biological principles concerning additive and replacing HGTs.

### Biological implications

The dominance of replacing HGT within species boundaries agrees with the proposal of Dykhuizen and Green (1991) that replacing HGT followed by recombination can be used to define species similar to the biological species concept. However, integration into a genome through homologous recombination requires only short stretches of similar sequences flanking the recombining DNA (Lorenz and Wackernagel 1994). Consequently, in bacteria and archaea, replacing HGT is not limited by a well-defined species boundary, rather a steep gradient for the successful integration of transferred genetic material exists (Roberts and Cohan 1993; Vulic et al. 1997; Williams et al. 2012). HGT between close relatives followed by homologous recombination, similar to eukaryotic sex, counteracts the accumulation of slightly deleterious mutations; however, replacing HGT also prevents or at least slows down the co-evolution of genes. The complexity hypothesis (Jain et al. 1999) suggests that genes, whose products are part of large structurally integrated complexes, are only infrequently transferred. Our findings, and previous findings on the transfer of ribosomal RNAs and proteins (Mylvaganam and Dennis 1992; Ho et al. 1999; Gogarten et al. 2002; Zhaxybayeva et al. 2009) suggest that this might not always be the case. Further analyses based on larger data sets will be necessary to determine if the disruption of co-evolution slows down replacing transfers of genes encoding parts of a complex machinery.

The finding that additive HGTs generally occur across species boundaries agrees with the understanding of genomic islands as providing adaptation to a particular ecological niche (Dobrindt et al. 2004). This interpretation is also supported by many genes in the additive HGT category encoding proteins involved in the uptake and metabolism of substrates, which may help the recipient to adapt to a particular niche in which these substrates are available (Juhas et al. 2009), or rather these islands are adapted to an ecological niche and are transferred into organisms occupying or invading this niche (Papke and Gogarten 2012). The other large additive transfer category is, unsurprisingly, prophages and selfish genetic elements. Short deletions (Mira et al. 2001; Bobay and Ochman 2017) and recombination events that separate deleterious from advantageous genes (Nguyen et al. 2022) are ways that selfish genetic elements can be inactivated and lost from genomes. Finally, analysis of the divergence between Escherichia and Salmonella (Retchless and Lawrence 2007) suggests that additive transfers locally impact replacing transfers. An adaptive gene integrated into a genome through additive HGT can prevent replacing HGT from donors that do not harbor this gene because homologous recombination usually requires homologous flanking regions on both ends of integrated gene. Thus, in the immediate genome neighborhood of the added gene, a slowly expanding island is created in which polymorphisms accumulate preventing further homologous recombination.

### Impact of dataset construction choices on classification

Our analysis of the *Aeromonas* dataset identifies unrecognized homology as a key reason for potential misclassification by DART. Such unrecognized homology can result from incomplete gene calling during the annotation or over-splitting of gene families during the classification of homologous groups. Any high-throughput, automated pipeline for gene annotation and gene family clustering, such as RAST (Aziz et al. 2008) and OrthoMCL (Li et al. 2003) (plus single linkage clustering) as used in the construction of the *Aeromonas* dataset, is likely to be affected by this problem to at least some degree (see, e.g. Bakke et al. 2009). Thus, additional filtering of classification results using BLAST, as we demonstrate, may be necessary to validate the results when analyzing large datasets created using automated computational pipelines. In addition, as our case study of cHG 21,480 shows (supplementary Figure S12, Supplementary Material online), it may be beneficial to use multiple different annotation software when studying and interpreting specific HGTs, or even as a way to identify potential false-positive and/or false-negative classifications.

### Methodological limitations

The classification approach implemented in DART has two key limitations. First, DART can only classify HGTs that are between terminal edges, i.e. whose donor and recipient are along terminal edges on the species tree. This is because DART relies critically on gene ordering information for the recipient species, which is not directly available for ancestral species. The resulting focus on only HGTs between terminal edges can bias the study of replacing versus additive HGTs since the effect of selective pressures on recently horizontally acquired genetic material may not be fully accounted for. Thus, results related to relative frequencies of additive and replacing HGTs and to their functional biases should be interpreted with caution. Second, DART only classifies single-gene HGTs or HGTs that may be part of small HMGTs, not large HMGTs. This is due to DART’s reliance on assessing the similarities of the gene neighborhoods of the transferred gene on the recipient genome and its homologs on the closest phylogenetic neighbors, which can be misled by large HMGTs. In addition, as discussed above, DART’s classifications can be sensitive to incomplete gene calling or under-clustering of gene families. As we show in this work, it is possible to detect and filter out many potentially misclassified HGTs, but this reduces the number of HGTs ultimately classified. Such filtering also affects additive HGTs more than it affects replacing HGTs, which can artificially lower the fraction of HGTs classified as additive post filtering.

At a more fundamental level, the accuracy of any HGT classification framework depends on the accuracy of HGT detection itself. While DART uses state-of-the-art phylogenetic approaches for HGT detection, these methods still suffer from relatively high false-positive and false-negative inference rates (Nguyen et al. 2013; Bansal et al. 2015, 2018). DART’s focus on classifying only terminal-edge HGTs, along with its use of conservative HGT inference parameters, helps minimize the impact of false-positive inferences, but still, at least some of the classified “HGTs” are likely to either not be true HGTs or have their donors and/or recipients incorrectly inferred. The potentially large false-negative rate for the conservative HGT inference pipeline used by DART can also be problematic in that it can lead to biases in the kinds of HGTs that are detected, potentially leading to biased down-stream inferences when analyzing the classified additive and replacing HGTs. Moreover, it can be very difficult, or even impossible, to detect many HGTs between closely related species or strains due to insufficient sequence divergence, which can again potentially bias downstream inferences.

### Future directions

Several aspects of the classification approach implemented in DART can be further improved. For instance, it may be possible to overcome the restriction to classifying only terminal-edge HGTs by combining DART with methods that can infer ancestral gene content and ordering (Duchemin et al. 2017; Feng et al. 2017). DART could then be applied to all HGTs for which gene contents and orderings of their recipients can be inferred with reasonable certainty. It would also be worth extending DART to classify inferred HMGTs (Kloub et al. 2021). The existing algorithms implemented in DART can easily be used to classify HMGTs if the boundaries of an HMGT are inferred accurately, However, due to HGT inference error and uncertainty, it is often difficult to infer the precise breakpoints for an HMGT. Nonetheless, by using sequence-based features such as GC content or codon usage bias to identify HMGT breakpoints, together with better accounting for breakpoint uncertainty in GNC calculations, it may be possible to reliably classify recent HMGTs as being additive or replacing. Finally, it would be very interesting to combine recently developed, but as yet unreliable, reconciliation-based approaches for classifying additive and replacing HGTs (Kordi et al. 2019; Mondal et al. 2020). By combining DART with such reconciliation-based approaches, it may be possible to both improve classification accuracy and classify a larger fraction of HGTs.

## Materials and Methods

### *Aeromonas* Dataset

For our experimental analysis, we used the 103-genome *Aeromonas* dataset recently described by Kloub et al. (2021) and constructed using previously published genomes Rangel et al. (2019). This dataset consists of 103 *Aeromonas* genomes/strains representing 28 distinct species. Among these, 18 species are represented by a single genome, and 10 are represented by multiple genomes. As detailed in Kloub et al. (2021), protein coding ORFs were called on these genomes using the RAST annotation server (Aziz et al. 2008). This resulted in an average of ∼ 4,090 genes per genome. For reference, a complete listing of *Aeromonas* genomes used, along with statistics on genome completeness, GC content, and genome size appear in supplementary Table S14, Supplementary Material online of Kloub et al. (2021). Notably, the 103 genomes have an average completeness score of 99.47%, and only one genome has a completeness score below 97.9%. The genome assemblies have an average of 93.5 contigs, with a median of 67. Only 23 genomes have more than 100 contigs. Note that plasmids simply appear as contigs in our dataset and DART does not distinguish between plasmids and other contigs. If desired, users of DART may choose to remove plasmids from the genome data provided as input to the tool.

**Fig. 6.**
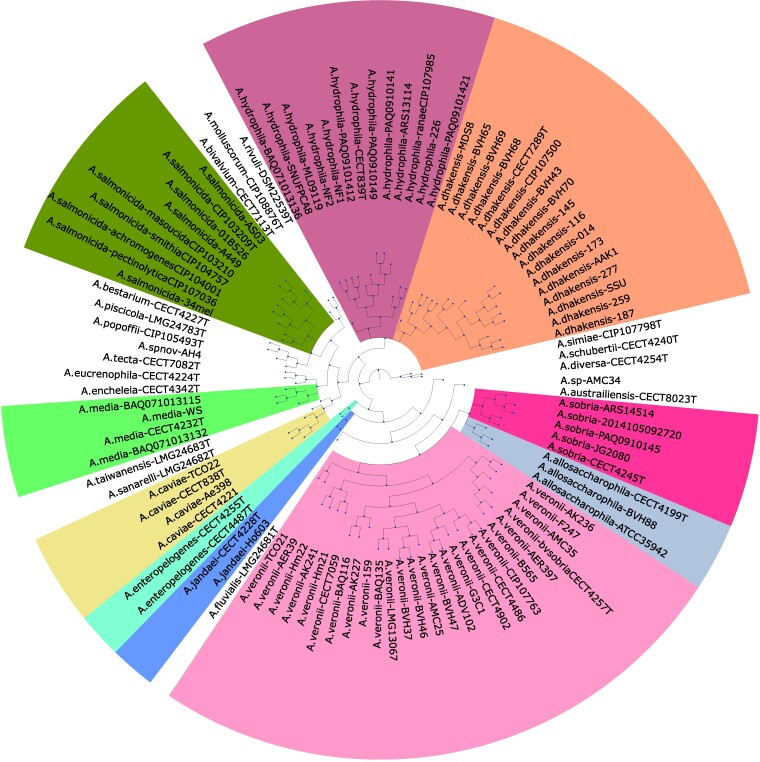
The 103-genome *Aeromonas* species tree (topology only) used in this work. Strains belonging to the same species are all assigned the same (nonwhite) color.

In addition to the 103 genomes and their ORFs/genes, the dataset from Kloub et al. (2021) consists of (i) A rooted species tree inferred via the 16-gene multilocus sequence analysis (MLSA) scheme previously established for use in the *Aeromonas* (Colston et al. 2014), reconstructed using RAxML (Stamatakis 2014) (GTR+GAMMA+I model), and rooted along the branch connecting the *A. schubertii, A. diversa, A. simiae* clade to the rest of the tree (Colston et al. 2014; Rangel et al. 2019). This 16-gene MLSA approach has been shown to be comparable to more complicated methods such as expanded core MLSA and whole-genome methods (Colston et al. 2014; Gosselin et al. 2021). This species tree is shown in Fig. 6. A version of this tree with branch lengths and bootstrap support values is shown in supplementary Figure S13, Supplementary Material online. (ii) 22282 consolidated homologous groups (cHGs), i.e. gene families, with functional annotations available for each. The cHGs were obtained by first clustering genes using OrthoMCL (Li et al. 2003), as implemented in the GET_HOMOLOGUES software package (Contreras-Moreira and Vinuesa 2013), and then further consolidating/merging clusters that showed high similarity between their longest genes/ORFs using single-linkage clustering (see Kloub et al. 2021 for details). Functions were assigned to each cHG by querying sequences against the Trinotate customized version of the Pfam database (Bryant et al. 2017). And (iii) high quality gene trees for all 7,567 cHGs containing at least four genes, constructed using RAxML (Stamatakis 2014) and further error-corrected using TreeFix-DTL (Bansal et al. 2015). Given the high level of similarity between the genomes (average ANI of 88.78%), the alignments used for gene tree construction were computed using MUSCLE (v3.8.31) (Edgar 2004). Both RAxML and TreeFix-DTL were run using the GTR+GAMMA+I model and thorough search settings (see Kloub et al. 2021 for details). These 7,567 gene trees have, on average, 53.1 leaves. Genes present in the remaining cHGs, i.e. those containing only 1, 2, or 3 genes, were not analyzed since they either represent trivial classification instances (e.g. all genes in cHGs containing only one gene would be trivially classified as additive, as would those genes in cHGs containing two or three genes that are “distant” from their other homolog(s) on the species tree) or are acquired by HGT to an ancestral (nonleaf) species. Such genes were aggregated into a list of “rare” genes and used as part of the HMGT filtering step as described later in this section.

### Methodological Details

We describe the key steps of DART in detail below.

#### Inference of high-confidence terminal-edge HGTs

We used phylogenetic reconciliation of the gene trees with the species tree to infer HGTs. Specifically, we employed the Duplication-Transfer-Loss reconciliation framework, which explains gene tree/species tree discordance using gene duplication, HGT, and gene loss events, implemented in the popular RANGER-DTL 2.0 software package (Bansal et al. 2018) to both root the gene trees and infer HGT events. We used default costs for duplication and loss events (David and Alm 2011; Bansal et al. 2013), but a slightly higher cost for HGT events (cost four instead of the default cost of three) to better distinguish between possible gene duplication and HGT and minimize false-positive HGTs. To further account for HGT inference uncertainty and ambiguity of identifying the donor and recipient for an HGT event (Bansal et al. 2013), we ran RANGER-DTL 100 times for each gene tree and then aggregated across the resulting reconciliation to identify those HGTs that had 100% support (i.e. were inferred in all 100 reconciliation samples) and had at least 51% support for the donor and recipient assignment. Any HGT for which the donor or recipient did not occupy a terminal edge, i.e. a leaf-edge, of the species tree was discarded. This resulted in the identification of 53,615 highly supported terminal-edge HGTs.

#### Mapping inferred HGTs to genomic locations

Each gene in each genome is associated with a unique *gene ID*, which identifies that specific gene uniquely across all 103 genomes, as well as with a *cHG ID*, which specifies the cHG to which that gene belongs. In order to determine the gene neighborhood around the transferred gene, DART requires knowledge of the gene ID of the transferred gene in the recipient genome. However, since a genome may contain more than one gene from the same cHG, it can sometimes be difficult or impossible to use phylogenetic reconciliation to determine the specific gene sequence that was transferred (e.g. in case of gene duplication or subsequent HGT following an HGT event). Among the 53,615 inferred high-confidence terminal-edge HGTs, we were able to determine the specific donor and recipient gene IDs for 52,692 (or 98.2%). Among these, gene IDs for 51,170 HGTs could be directly identified since the donor and recipient genomes had only one gene from the corresponding cHG. Gene IDs for the remaining 1,522 HGTs could be identified by parsing through the reconciliations computed by RANGER-DTL. Of the 52,692 HGTs with known gene IDs, 39,048 are intra-species and the remaining 13,644 are inter-species.

#### Identifying and filtering out potential HMGTs

We further filtered the set of 52,692 high-confidence terminal-edge HGTs with known gene IDs to remove any potential HMGTs. We removed such potential HMGTs since it can often be difficult to precisely identify the end points of HMGT events (Kloub et al. 2021), making it difficult to confidently identify the neighboring genes of such transfers (i.e. gene neighbors that did not themselves get transferred as part of the HMGT). To identify potential HMGTs, we first identified an expanded set of potential HGT events by using more permissive HGT inference settings for RANGER-DTL. Specifically, we used a lower cost of three for HGT events and a lower support threshold for HGT identification (we inferred HGTs whenever the most frequent event type was labeled as an HGT among the 100 sampled reconciliations for a gene trees). If we could not identify the specific gene ID for a potential HGT, then we flagged each homolog of that transferred gene in that genome as a potential HGT.

Any HGT from our set of high-confidence terminal-edge HGTs with known gene IDs that either had (i) at least one potential HGT from the same donor (as identified using the permissive settings described above) among its four closest gene neighbors (i.e. two on either side) along the recipient genome , or (ii) at least two potential HGTs from the same donor among its *n* closest gene neighbors along the recipient genome, was flagged as a potential HMGT and removed from further consideration. Since rare genes (i.e. those present in cHGs containing only 1, 2, or 3 genes) are likely to have been acquired via HGT/HMGT from outside the species tree, potentially complicating our gene neighborhood conservation analysis, we also filtered out those HGT from our set of high-confidence terminal-edge HGTs with known gene IDs that had either at least one rare gene among the four closest gene neighbors or at least two rare genes among their *n* closest gene neighbors along the recipient genome. After applying this filtering step to the 52,692 high-confidence terminal-edge HGTs with known gene IDs, we were left with 40,521 high-confidence *classifiable* terminal-edge HGTs with known gene IDs. As noted previously, these “classifiable” HGTs consist of single-gene HGTs as well as, possibly, HGTs that are part of small undetected HMGTs. Small undetected HMGTs do not pose a problem for DART since they are highly unlikely to affect classification accuracy.

#### Assessing phylogenetic and genic neighborhood conservation

For each inferred high-confidence classifiable terminal-edge HGT, we quantified its phylogenetic neighborhood conservation (PNC) and gene neighborhood conservation (GNC) as follows. To compute the PNC, we first identify the *m* closest phylogenetic neighbors of the recipient species/strain on the species tree and then determine how many of these *m* phylogenetic neighbors have at least one homolog of the transferred gene. Let *R* denote the recipient genome and *G* denote another genome that contains a homolog of the transferred gene. Then, we define the GNC between *R* and *G*, with respect to the transferred gene, as follows: Given an even number *n*, we consider the two sets of nearest n/2 genes on either side of the transferred gene along the recipient genome. We then consider the two sets of nearest n/2 genes on either side of the of the homolog of the transferred gene along genome *G* and compute the greatest percent overlap between the two pairs of sets. For example, for n=8, if the two neighboring gene sets along *R* are A={1,2,3,4} and B={5,6,7,8}, and the two neighboring gene sets along *G* are C={1,2,3,5} and D={6,7,9,10}, then *A*’s best match is with *C* and their overlap is three genes (out of a maximum of four) and the best match for *B* is *D* with an overlap of two out of four genes. The total number of overlapping genes is therefore five, giving a GNC of (5/8)×100=62.5%. If *G* contains multiple homologs of the transferred gene then the maximum GNC across all homologs is used.

If the transfer gene in the recipient, or its homologs in the phylogenetic neighbors of the recipient genome appears near the end of contig where some of the neighboring genes are absent, then it may not be possible to compute GNC as described above. If the transferred gene in the recipient genome appears towards the end of a contig such that more than two genes out of the neighborhood of *n* genes are absent (i.e. cut off by a contig end), then that HGT is immediately classified as ambiguous. If only one or two genes in the neighborhood are absent, then the GNC is calculated using only the remaining n−1 or n−2 neighboring genes. If homologs of the transferred gene appear near contig ends in the phylogenetic neighbors, then any phylogenetic neighbor that has more than two of the neighboring genes of the homolog absent is discarded, effectively reducing the number of phylogenetic neighbors considered for the classification. If all *m* phylogenetic neighbors have homologs near contig ends, such that more than two of the neighboring genes are absent for each, then that HGT is classified as ambiguous.

#### Classification of HGTs as additive, replacing, or ambiguous

The classification of HGTs by DART is performed in accordance with Observations 1 and 2. To classify an HGT as additive, we verify that all of the recipient’s *m* closest phylogenetic neighbors either do not contain a homologous gene or contain homologous genes only in clearly nonconserved genomic contexts (i.e. meet the GNC threshold for nonconservation). Likewise, to classify an HGT as replacing, we require that at least some number of the *m* closest phylogenetic neighbors contain homologous genes in a conserved genomic context (i.e. exceeds the GNC threshold for conservation). The specific GNC threshold values used for determining conservation and nonconservation, as well as values of the other parameters relevant for classification are discussed in the subsection titled “Specific parameter choices” below. Any HGT that cannot be classified as either additive or replacing using the given parameter/threshold values is left unclassified.

#### Post-processing and further filtering of classification results

Observe that incomplete gene calling or potential under-clustering of gene families can lead to erroneous assessments of phylogenetic and genic neighborhood conservation, leading to incorrect HGT classification. To account for potential classification errors due to such data quality issues or other reasons, we perform additional analysis and filtering of DART’s classification results using BLAST, gene orderings for donor genomes, and other information. In particular, we (i) use BLAST to ensure that a particular genome indeed does not have any homolog of the transferred gene, (ii) perform more fine grained analysis of HGT cases where homologs in phylogenetic neighbors are close to contig ends, (iii) take into consideration neighboring genes that may be absent from the genomes of the phylogenetic neighbors of the recipient, indicating the presence of an undetected HMGT, and (iv) use knowledge of the gene ordering along the donor genome to help validate HGTs classified as replacing. Post-processing Steps (i) and (ii) can be used to identify possible HGTs whose classification as additive may be erroneous or not well supported. Step (iii) can be used to detect possible cases of undetected HMGT that may be affecting classification results. And Step (iv) can be used to identify possible HGTs whose classification as replacing may be erroneous or not well supported. These post-processing analyses are described in detail in the Results section.

### Specific Parameter Choices

Once high-confidence classifiable terminal-edge HGTs have been inferred, the most important parameters impacting the classification are (i) the number of closest phylogenetic neighbors, *m*, considered when calculating the PNC, (ii) the size of the gene neighborhood, *n*, used when computing GNC, (iii) the specific classification thresholds for PNC and GNC used to classify HGTs as additive, and (iv) the specific classification thresholds for PNC and GNC used to classify HGTs as replacing. We describe our choices for these parameters in detail below.

#### Number of phylogenetic neighbors

To conservatively assess the presence of homologs in strains/species closely related to the recipient, we considered the three closest phylogenetic neighbors of the recipient genome on the species tree, i.e. we used m=3 by default. Note that if one of these three closest phylogenetic neighbors happened to be the donor of the transferred gene, then we skipped over the donor and chose the next closest phylogenetic neighbor instead. We also considered the impact of reducing this parameter value to 1 and 2.

#### Size of gene neighborhood

To adequately assess conservation of genomic context, the size of the gene neighborhood used to compute GNC should be neither too small, which would result in excessive sensitive to small changes in the gene neighborhood, nor too large, which would increase susceptibility to gene content variation, rearrangements, etc., between the genomes. We therefore chose n=8 as our default value for the gene neighborhood size parameter. Specifically, this involves considering the four nearest genes before and the four nearest genes after the transferred gene along the recipient genome. To assess the impact of this parameter in practice, we also considered the parameter values n=4 and n=16.

#### Classification thresholds for additive HGTs

By default, we use conservative PNC and GNC thresholds to classify HGTs as additive. In particular, to classify an HGT as additive, we require all of the recipient’s *m* closest phylogenetic neighbors to either not contain a homologous gene or contain homologous genes only in clearly nonconserved genomic contexts (i.e. meet the GNC threshold for nonconservation). The default GNC threshold for inferring that the gene neighborhood is nonconserved is 20%. For our default gene neighborhood size of n=8, this means that at most 1 out of the 8 genes can be conserved across the gene neighborhoods being compared. To assess the impact of this GNC threshold on classification, we also considered 0%, 10% and 30% thresholds.

#### Classification thresholds for replacing HGTs

As with additive HGTs, by default, we use conservative thresholds to classify HGTs as replacing. Specifically, we require that at least 1 of the *m* closest phylogenetic neighbors contain homologous genes in a conserved genomic context (i.e. exceeds the GNC threshold for conservation). The default GNC threshold for inferring that the gene neighborhood is conserved is 80%. For our default gene neighborhood size of n=8, this means that at least seven out of the eight genes must be conserved across the gene neighborhoods being compared. To assess the impact of this GNC threshold on classification, we also considered 70%, 90%, and 100% thresholds.

### Statistical Analysis

We used statistical analysis to estimate false-positive rates for our classification, i.e. the fraction of classified HGTs expected to be false positives. Specifically, we estimated rates of classifying a replacing HGT as an additive HGT, and of classifying an additive HGT as a replacing HGT. We computed these false-positive rates for different combinations of parameter settings.

To determine how often a replacing HGT may get classified as an additive HGT, we considered the null hypothesis that all HGTs are replacing and then estimated the fraction of those HGTs that would be classified as additive using DART with specific parameter settings. To perform this estimation, we repeatedly generate a randomized set of HGTs (all assumed to be replacing) that preserves the overall phylogenetic distribution, counts, and characteristics of the inferred HGTs, and apply DART to these randomized collections of HGTs to determine the fraction of the randomized HGTs that DART infers as being additive. Specifically, for each pair of donor–recipient edges $\left(e_{i},e_{j}\right)$ on the species tree, let $H_{ij}$ denote the set of inferred single-HGT events inferred with $e_{i}$ as donor and $e_{j}$ as recipient. Let $\left(e_{k},e_{l},e_{m}\right)$ denote the three closest phylogenetic neighbors of the recipient genome on the species tree. Let $G_{\;jklm}$ denote the set of cHGs common to all the species represented by edges $e_{j}$, $e_{k}$, $e_{l}$, and $e_{m}$. Then, we randomly choose $\left|H_{ij}\right|$ genes from these gene families along the recipient genome to be the new, randomized set of HGTs between $\left(e_{i},e_{j}\right)$. We then execute DART, as usual, using these randomized HGTs. Based on the specific selection process described above, we expect most of the randomized “HGTs” to be replacing (since their homologs appear in all *m* phylogenetic neighbors). The number of such randomized HGTs classified as additive thus provides an estimate of the false-positive rate for classifying a replacing HGT as an additive HGT. To obtain a reliable estimate of this rate, we perform 100 such randomizations and average over the results.

To determine how often an additive HGT may get classified as a replacing HGT, we considered the null hypothesis that all HGTs are additive and then estimated the fraction of those HGTs that would be classified as replacing using DART with specific parameter settings. We use a similar randomization-based approach to estimate this false-positive rate. Specifically, we used the following randomization procedure. Let $e_{i}$, $e_{j}$, $H_{ij}$, and $e_{k},e_{l},e_{m}$ be as defined above. Let $A_{\;j}$, $A_{k}$, $A_{l}$, and $A_{m}$ denote the set of cHGs from the genomes represented by edges $e_{j}$, $e_{k}$, $e_{l}$, and $e_{m}$. Then, we randomly choose $\left|H_{ij}\right|$ genes from $A_{j}$ along the recipient genome to be the new, randomized set of HGTs between $\left(e_{i},e_{j}\right)$ but choose $\left|H_{ij}\right|$ genes at random from each of $A_{k}$, $A_{l}$, and $A_{m}$ to represent the “homologs” of the HGTs chosen on the recipient genome. We then execute DART, as usual, using these randomized HGTs and their randomized homologs. Based on the specific selection process described above, we expect most of the randomized “HGTs” to be additive given their randomly chosen homologs in the phylogenetic neighbors (since the genomic context of these “homologs” in the phylogenetic neighbors is expected to be different than in the recipient genome). The number of such randomized HGTs classified as replacing thus provides an estimate of the rate of classifying an additive HGT as a replacing HGT. As before, to obtain a reliable estimate of this false-positive rate, we perform 100 such randomizations and average over the results.

Note that the above description assumes that m=3. The randomization is performed analogously for other values of *m*.

### Scalability of the DART Framework

DART takes as input a collection of gene trees, a rooted species tree, and gene orderings for each genome included in the analysis. DART first reconciles the gene trees with the species tree to infer high-confidence classifiable terminal-edge HGTs. This step has a total time complexity of $O(ckn^{2})$, where *n* is the number of genomes in the analysis, *k* is the number of gene trees, and *c* is the number of reconciliation samples to be computed per gene tree. For our *Aeromonas* dataset with 7,567 gene trees, the time needed to compute 100 reconciliation samples for each gene tree, and repeated for two different transfer costs, was 144 h (6 days) using a single core of a 2.1 GHz Intel Xeon processor. The memory requirement for this step was less than 1 GB. Once the list of high-confidence classifiable terminal-edge HGTs has been computed, the core DART classification framework is highly efficient. On the *Aeromonas* dataset, DART was able to classify the over 40,000 HGTs within 42 min using a single-core of a 2.1 GHz Intel Xeon processor and required less than 1 GB of main memory.

## Supplementary Material

evae180_Supplementary_Data

## Data Availability

The genomic data (i.e. complete and draft *Aeromonas* genomes) underlying this article are all publicly available (Rangel et al. 2019). The gene families, gene trees, gene ordering information, species tree, and software used for our analysis are freely available from https://compbio.engr.uconn.edu/software/dart/.
