# Supplementary material for: Investigating Additive and Replacing Horizontal Gene Transfers Using Phylogenies and Whole Genomes
Source: Genome Biol Evol. 2024 Aug 20;16(9):evae180. doi: 10.1093/gbe/evae180 (PMC11375855; doi:10.1093/gbe/evae180)
Supplement: evae180_Supplementary_Data [file evae180_supplementary_data.pdf]

Investigating Additive and Replacing Horizontal Gene Transfers Using Phylogenies and Whole Genomes: Supplementary Material

Kloub, Gosselin, Graf, Gogarten, and Bansal

Supplementary Text

Prokka Annotations and Gepard Plots

The Prokka Annotations and Gepard Plots shown in Supplementary Figures S8, S11, and S12 were obtained as follows. First, genome fragments around the site of transfer were re-annotated using Prokka v1.14-dev (Seemann 2014) with options: –kingdom Bacteria, –genus aeromonas. Each new annotation was also assigned new locus tags associated with the genome in question. These re-annotated fragments were aligned using the GEnome PAir Rapid Dotter (Gepard v1.40) (Krumsiek et al. 2007) with default parameters to produce Gepard plots. Plots were exported, and arrows indicating annotated genes were added manually.

The figure indicating the differences in annotation between RAST and Prokka (Supplementary Figure S12) was created using the annotations from RAST (from our main pipeline), and those created from Prokka (as shown above). These were fed through in house perl scripts to extract the locations of annotated genes along contig 17, and this data table was fed into R. R packages ggplot2 (v3.3.5) (Wickham 2016), gggenes (v0.4.1) (Wilkins and Kurtz 2020), RColorBrewer (v1.1-2) (Neuwirth 2014), and gghighlight (v0.3.2) (Yutani 2021) were used to create partial figures, which were then manually tiled and styled. Code for the gene plots can be found on github: `https://github.com/SophiaGosselin/Kloub-et-al-2021`.

Supplementary Figures

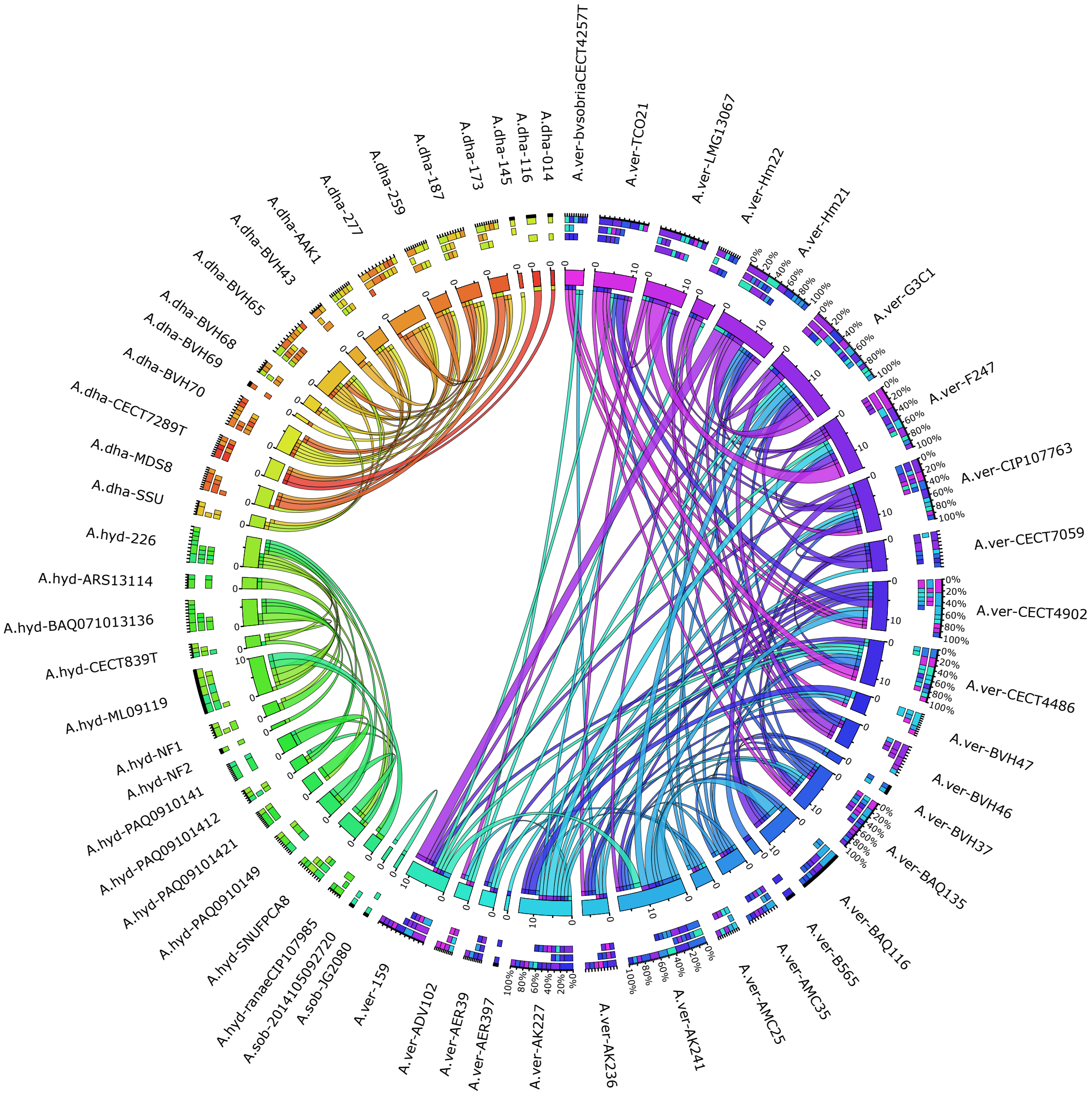

Figure S1. Intra-species additive HGTs. Each edge connects two *Aeromonas* genomes from the same species and corresponds to inferred intra-species additive HGTs between those two genomes. Edges are colored according to the color of the donor genome (the color for each genome is shown on the associated segment in the inner ring). The tip of a edge at the donor end is colored according to the recipient genome's color. The thickness of a edge corresponds to the number of additive HGTs for that donor-recipient pair, as quantified by the numbers around each segment in the inner ring. For each genome, both incoming (where that genome serves as recipient) and outgoing (where that genome serves as donor) edges are shown. The outer ring shows three stacked columns for each genome. Among these three stacked columns, the inner column shows the color distribution of recipients for outgoing edges, the middle column shows the color distribution of donors for incoming edges, and the outer column shown the combined color distribution for both incoming and outgoing edges, for that genome.

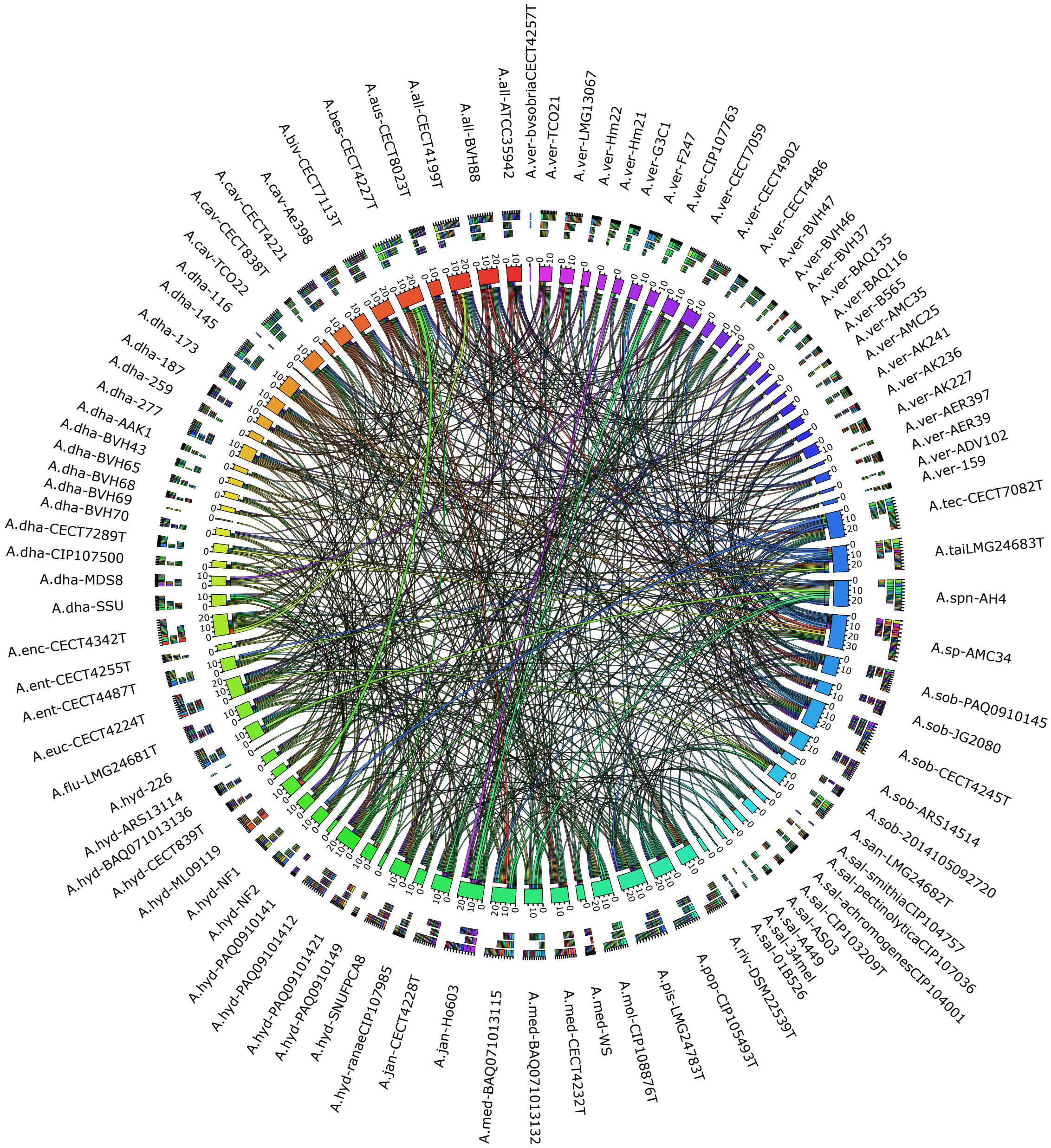

Figure S2. Inter-species additive HGTs. Each edge connects two *Aeromonas* genomes from different species and corresponds to inferred inter-species additive HGTs between those two genomes. Edges are colored according to the color of the donor genome (the color for each genome is shown on the associated segment in the inner ring). The tip of a edge at the donor end is colored according to the recipient genome's color. The thickness of a edge corresponds to the number of additive HGTs for that donor-recipient pair, as quantified by the numbers around each segment in the inner ring. For each genome, both incoming (where that genome serves as recipient) and outgoing (where that genome serves as donor) edges are shown. The outer ring shows three stacked columns for each genome. Among these three stacked columns, the inner column shows the color distribution of recipients for outgoing edges, the middle column shows the color distribution of donors for incoming edges, and the outer column shown the combined color distribution for both incoming and outgoing edges, for that genome. To discern individual edges, the figure must be viewed magnified on screen.

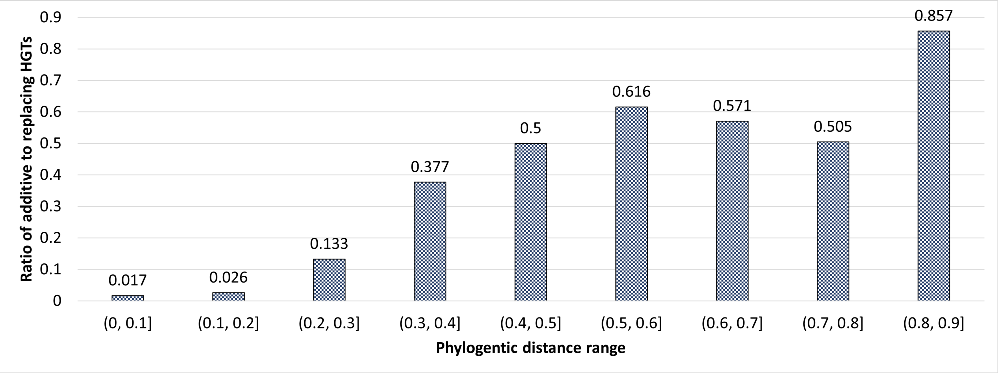

Figure S3. Fraction of unfiltered additive HGTs by phylogenetic distance. The plot shows the fraction of HGTs classified as additive for donor-recipient pairs separated by different phylogenetic distance ranges. Results are shown for the combined set of full, unfiltered inter- and intra-species HGTs classified as additive and replacing using default parameters. The phylogenetic distance between any donor-recipient pair is the patristic distance (with branch lengths representing substitutions per site) between the two corresponding terminal taxa on the species tree.

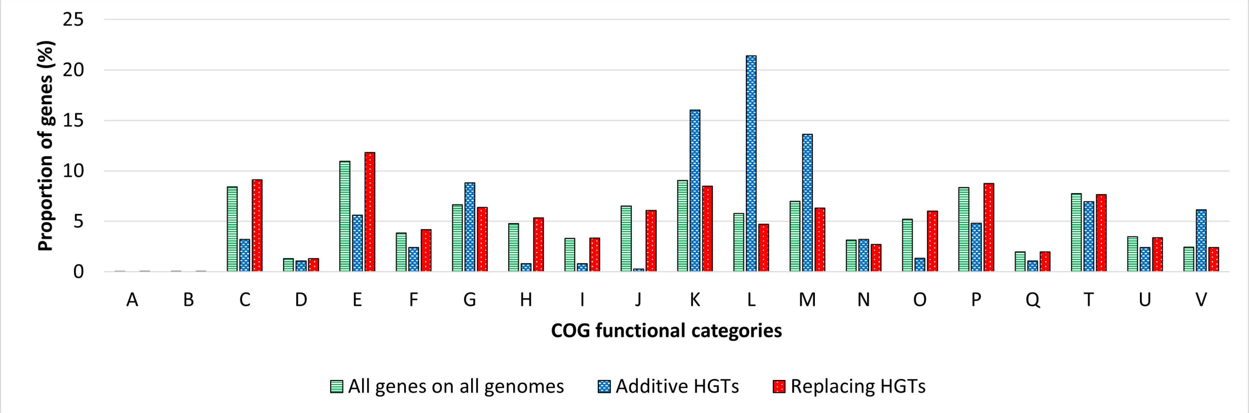

Figure S4. Functional analysis of additive and replacing HGTs. The figure shows distributions of COG categories with known functions for (i) all genes from all genomes, (ii) all HGTs classified as additive, (iii) all HGTs classified as replacing. Only HGTs present in the filtered classification results were used. Each letter corresponds to a COG functional category as shown in Supplemental Table S1. COG functional categories "Z", "Y", "W", and "R" are not shown since no gene in any of the *Aeromonas* genomes belonged to those categories. Only HGTs that could be assigned to a COG category with known function were considered in this analysis, i.e., HGTs that were assigned to category "S" or which could not be assigned to any COG category were excluded from this plot.

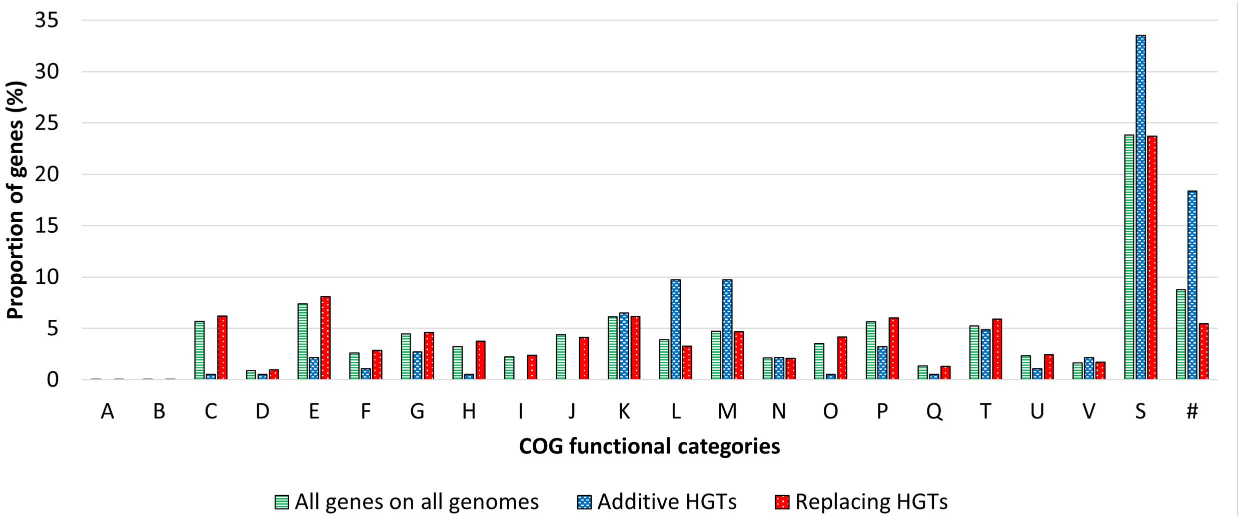

Figure S5. Functional analysis of intra-species additive and replacing HGTs. The figure shows distributions of COG functional categories for (i) all genes from all genomes, (ii) all intra-species HGTs classified as additive, (iii) all intra-species HGTs classified as replacing. Only HGTs present in the filtered classification results were used. Each letter corresponds to a COG functional category as shown in Supplemental Table S1. COG functional categories "Z", "Y", "W", and "R" are not shown since no gene in any of the *Aeromonas* genomes belonged to those categories. COG Functional category "S" corresponds to genes whose functions are unknown, while the category "#" corresponds to genes which could not be assigned to any COG functional category.

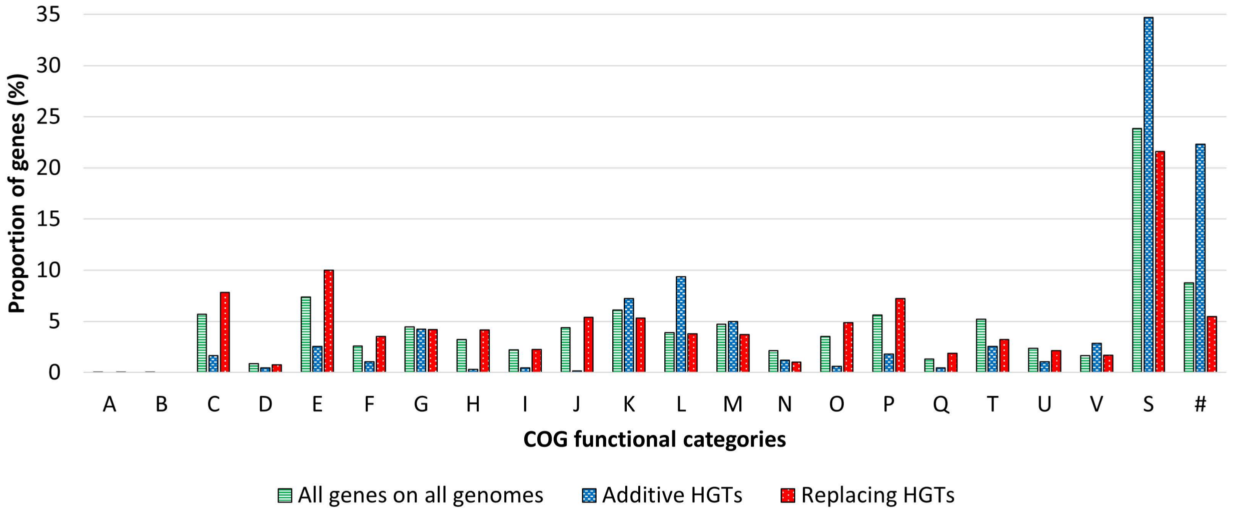

Figure S6. Functional analysis of inter-species additive and replacing HGTs. The figure shows distributions of COG functional categories for (i) all genes from all genomes, (ii) all inter-species HGTs classified as additive, (iii) all inter-species HGTs classified as replacing. Only HGTs present in the filtered classification results were used. Each letter corresponds to a COG functional category as shown in Supplemental Table S1. COG functional categories “Z”, “Y”, “W”, and “R” are not shown since no gene in any of the *Aeromonas* genomes belonged to those categories. COG Functional category “S” corresponds to genes whose functions are unknown, while the category “#” corresponds to genes which could not be assigned to any COG functional category.

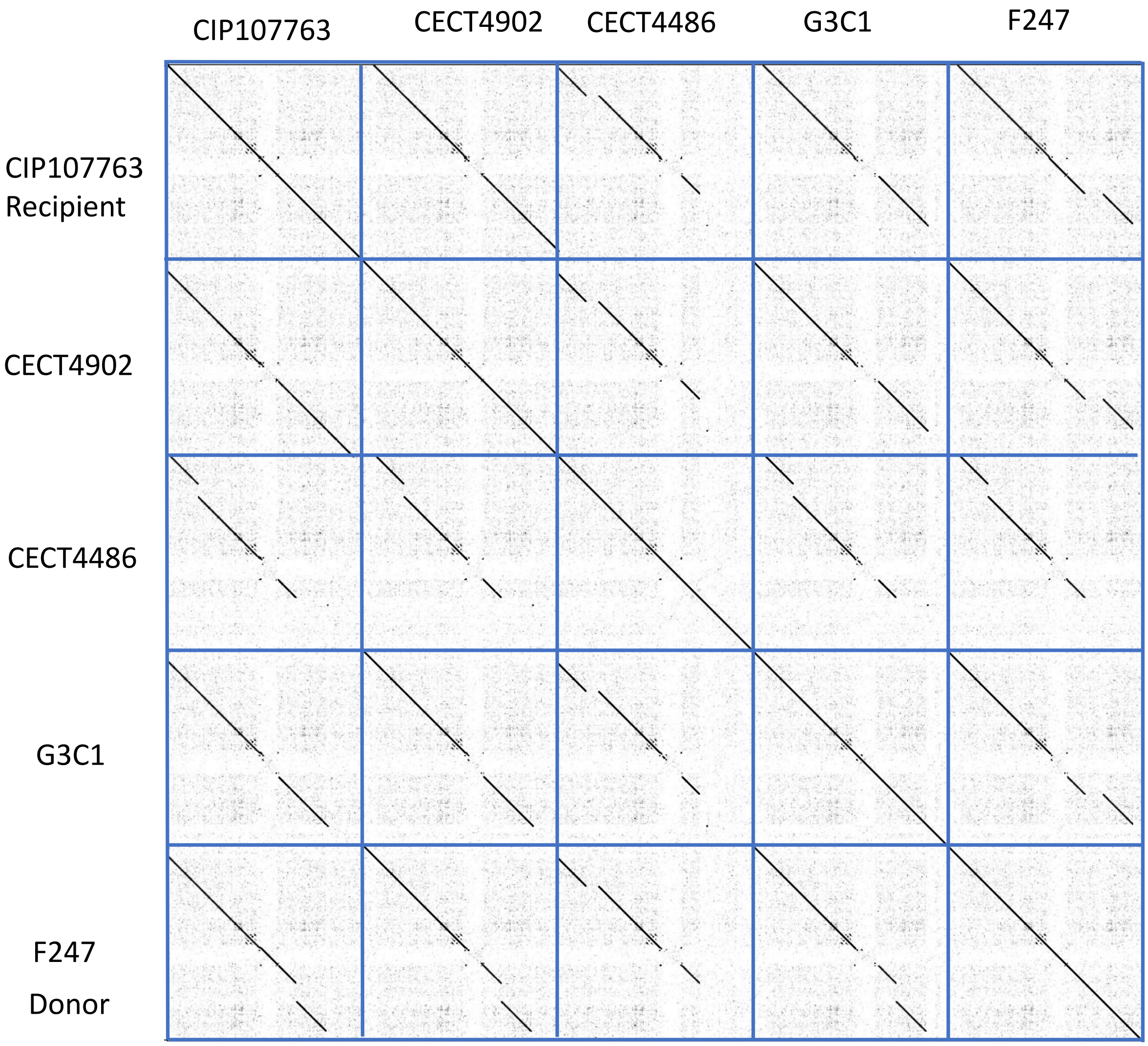

Figure S7. Dot plots of cHG 18292 transfer. Pairwise comparison of the donor (top row and left column), recipient (bottom row and right column) and all three neighboring genomes for the transfer of cHG 18292 from *Aeromonas veronii* F247 to *Aeromonas veronii* CIP107763. Note the gap along the alignment when comparing CIP107763 against its neighbors (the site of transfer) and the nearby region of repetitive DNA (possible hairpin loop site).

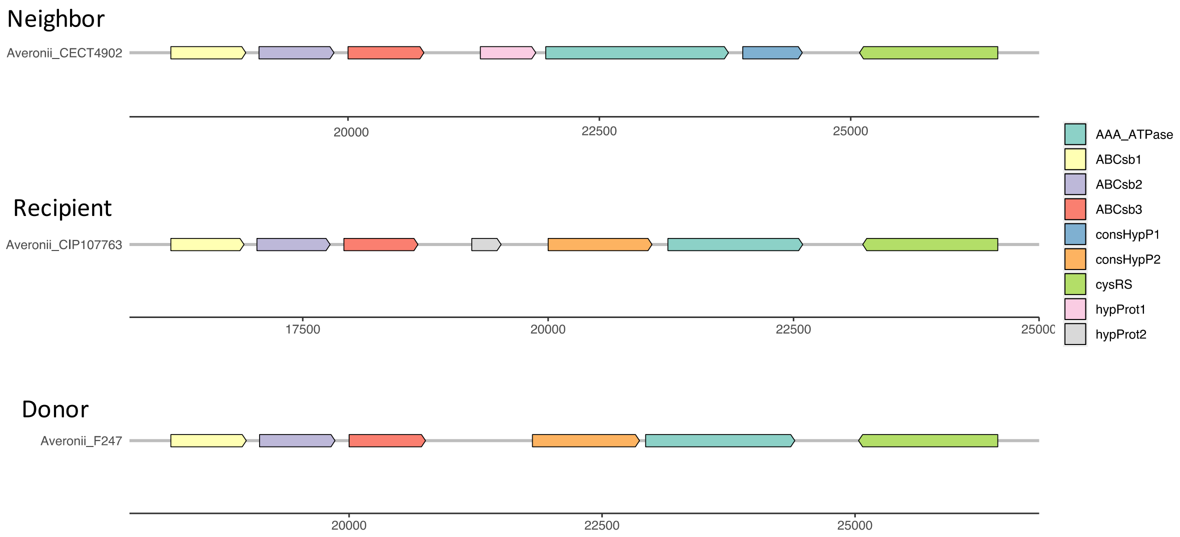

Figure S8. Gene plot of cHG 18292 transfer. Annotations shown are derived from Prokka. Coding direction is shown by arrow heads, and scale bars indicates the relative position in nucleotides along the contig. The region depicted here surrounds the region of low similarity between recipient and neighbor shown in Figure S7. The region of transfer is located between the three genes annotated as encoding substrate binding proteins of ABC transporters on the left and the cysRS encoding gene on the right. Abbreviations: AAA-ATPase, member of the AAA-ATPase family; ABCsb, ABC transporter substrate binding protein; consHypP, conserved hypothetical protein; cysRS, cysteinyl-tRNA synthetase; hypProt, hypothetical protein.

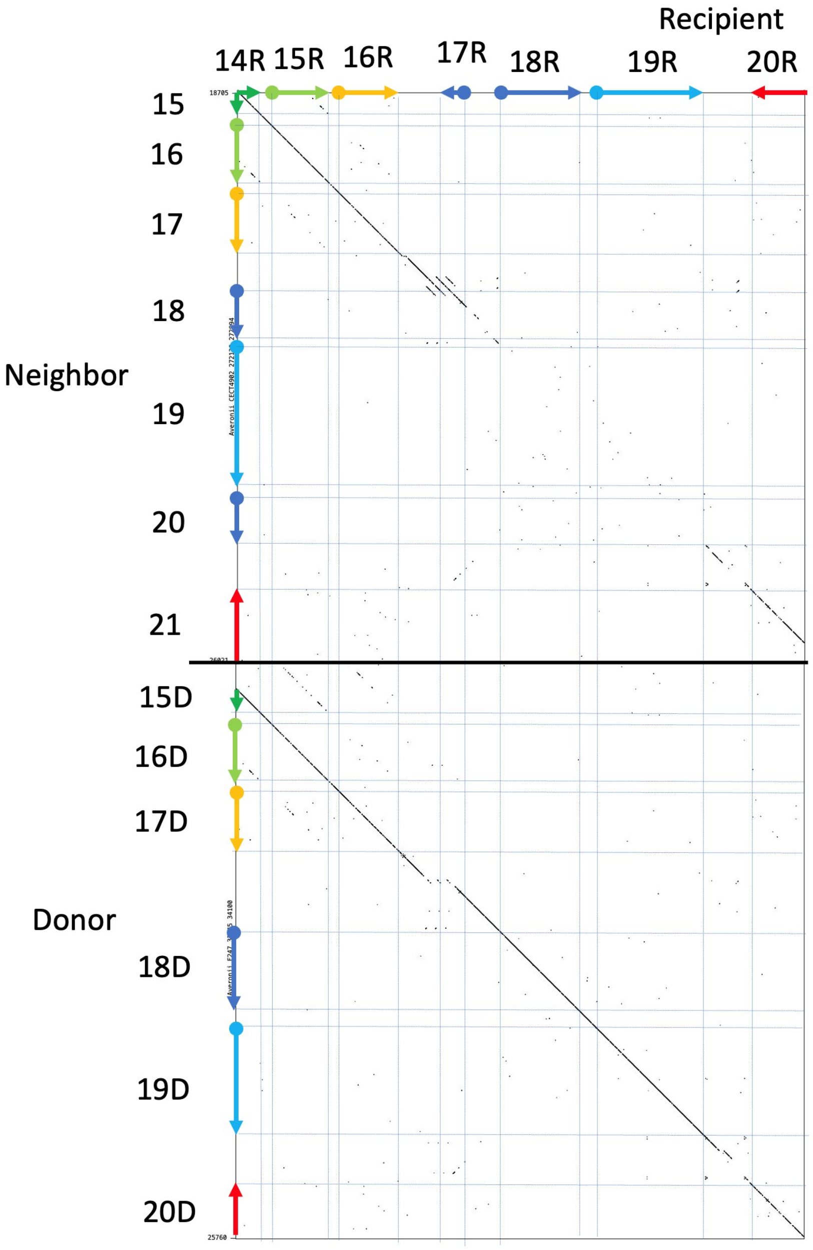

Figure S9. Dot plots of cHG 18292 transfer between Recipient and Neighbor (top) and Recipient and Donor (bottom). Annotations are from PROKKA (Seemann 2014), BLAST via the HHPred server using uniref 90, and HHPRED (Gabler et al. 2020). *Neighbors:* 15, 16, and 17: ABC Transporter Substrate binding domain containing protein (PBPb domain-containing). 18: uncharacterized protein – 2 matches in Aeromonas, HHPRED no significant match. 19: Homology to AAA family ATPases and nucleotide binding domains. 20: uncharacterized protein in Aeromonas, Vibrio, Plesiomonas, Photobacterium and Pseudomonas (BLAST). 21: Cysteine–tRNA ligase. *Recipient:* 14R, 15R, and 16R: ABC Transporter Substrate binding domain containing protein (PBPb domain-containing). 17R: Hypothetical protein – BLAST matches only in Aeromonads. 18R: Hypothetical protein, with homologs in cyanobacteria, gamma proteobacteria, planctomycetes); HHPRED: CoiA; Competence protein CoiA-like family. 19R: Homology to AAA family ATPases and nucleotide binding domains. 20R: Cysteine–tRNA ligase. *Donor:* 15D, 16D, and 17D: ABC Transporter Substrate binding domain containing protein (PBPb domain-containing). 18D: Hypothetical protein, with homologs in cyanobacteria, gamma proteobacteria, planctomycetes); HHPRED: CoiA; Competence protein CoiA-like family. 19D: Homology to AAA family ATPases and nucleotide binding domains. 20D Cysteine–tRNA ligase. While the AAA-ATPases family homologs in donor and neighbor do not reveal similarity in the dot plot, in PRSS the encoded proteins in a pairwise comparison had a z-score of 139.8 and an E(10000) value of 0.058 (Pearson 1996), revealing replacement by a divergent homolog.

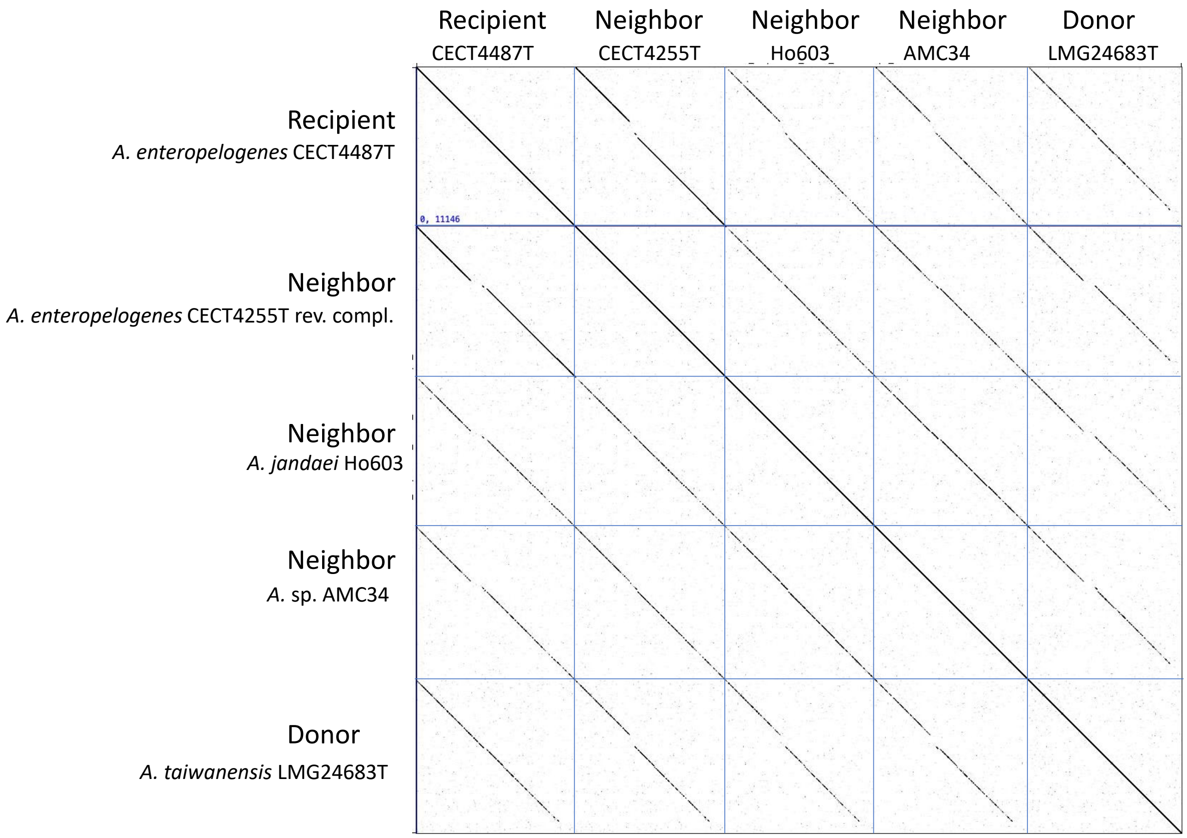

Figure S10. Dot plot of cHG 21480 transfer. Pairwise comparision of the donor, recipient and all three neighboring genomes for the transfer of cHG 21480 from *Aeromonas taiwanensis* LMG24683T to *Aeromonas enteropelogenes* CECT4487T. Note the small gap along the alignment when comparing CECT4487T against any sequence except the donor. This is the location of the additive transfer.

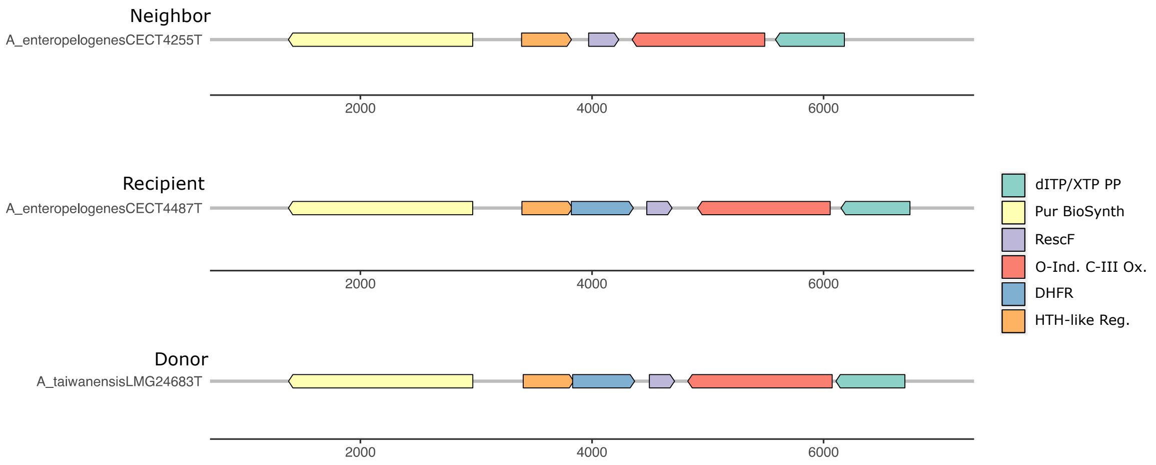

Figure S11. Gene plot of cHG 21480 transfer. Annotations shown are derived from Prokka. Coding direction is shown by arrow heads, and scale bars indicate the relative position in nucleotides along the associated fragment. The region of transfer is located between the genes annotated as Bifunctional purine biosynthesis protein on the left and the oxygen-independent coproporphyrinogen-III oxidase-like protein encoding gene on the right. Abbreviations: dITP/XTP pyrophosphatase: dITP/XTP PP, Bifunctional purine biosynthesis protein: Purine Biosynth, HTH-type transcriptional regulator: HTH-like Regulator, Dihydrofolate reductase: DHFR, Alternative ribosome-rescue factor A: RescF, O- Oxygen-independent coproporphyrinogen-III oxidase-like protein: O-Ind. C-III Ox.

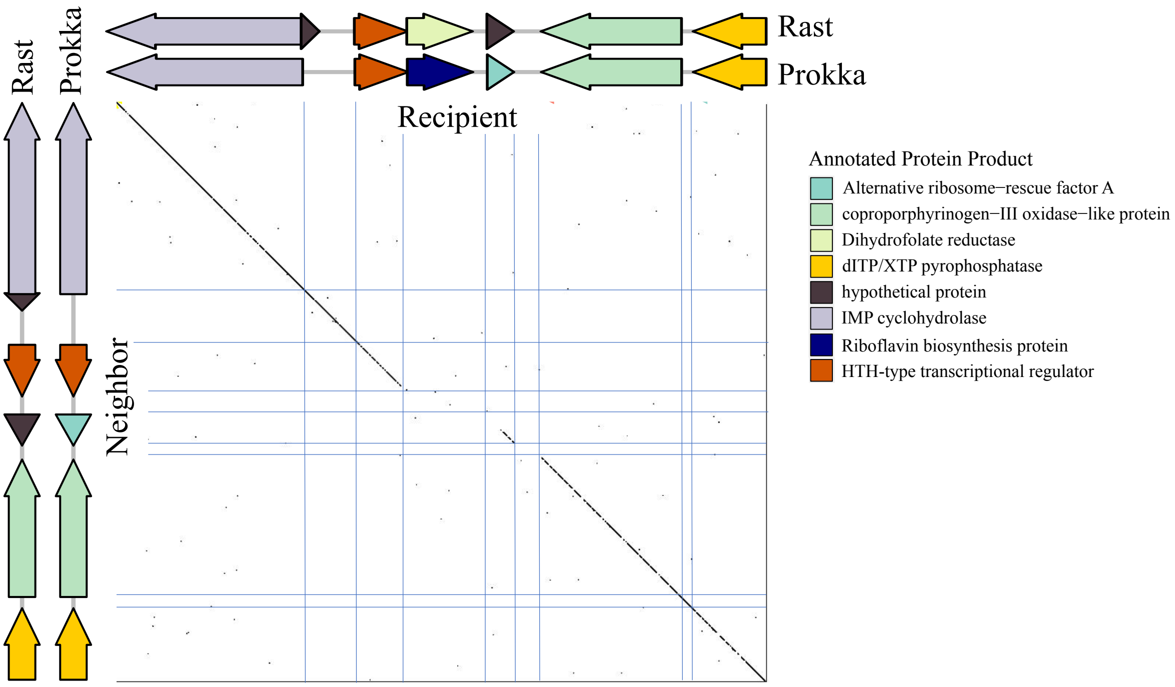

Figure S12. Dot plot of cHG 21480 transfer with annotations shown on the axes of the plot. Coding direction is shown by arrow heads, and annotations are from RAST and Prokka. The region of transfer is located between the genes annotated as IMP cyclohydrase and the oxygen-independent coproporphyrinogen-III oxidase-like protein. Note the difference in annotations between the RAST and Prokka annotations.

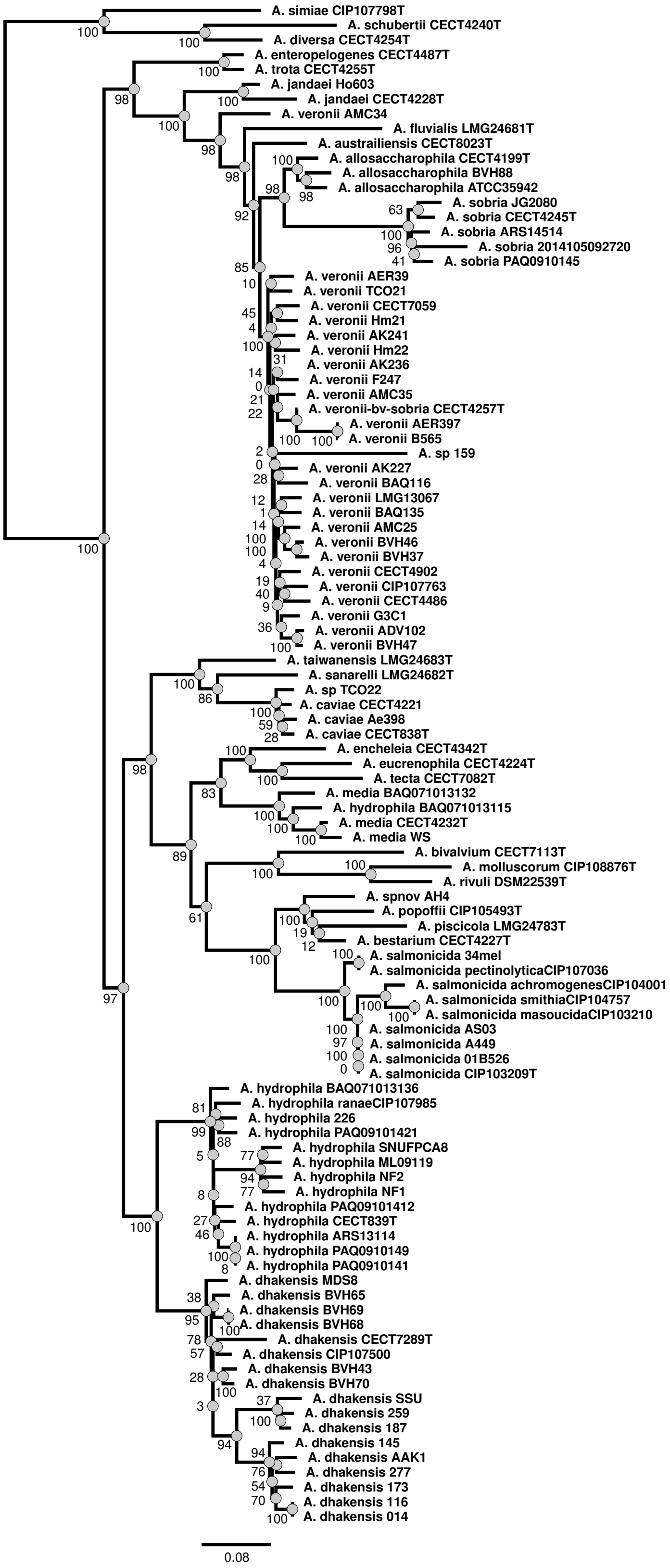

Figure S13. The 103-genome Aeromonas species tree with branch lengths and bootstrap support values. The tree was inferred using the 16 housekeeping gene multi-locus sequence analysis (MLSA) scheme previously established for use in the Aeromonas by Colston et al. (2014). Bootstrap support values were inferred from 100 replicates computed using the rapid bootstrap algorithm implemented in RAxML.

Supplementary Tables

Table S1. COG functional categories used for the functional analysis; see, e.g., Tatusov et al. (2000).

INFORMATION STORAGE AND PROCESSING
- [J] Translation, ribosomal structure and biogenesis
- [A] RNA processing and modification
- [K] Transcription
- [L] Replication, recombination and repair
- [B] Chromatin structure and dynamics

CELLULAR PROCESSES AND SIGNALING
- [D] Cell cycle control, cell division, chromosome partitioning
- [Y] Nuclear structure
- [V] Defense mechanisms
- [T] Signal transduction mechanisms
- [M] Cell wall/membrane/envelope biogenesis
- [N] Cell motility
- [Z] Cytoskeleton
- [W] Extracellular structures
- [U] Intracellular trafficking, secretion, and vesicular transport
- [O] Posttranslational modification, protein turnover, chaperones

METABOLISM
- [C] Energy production and conversion
- [G] Carbohydrate transport and metabolism
- [E] Amino acid transport and metabolism
- [F] Nucleotide transport and metabolism
- [H] Coenzyme transport and metabolism
- [I] Lipid transport and metabolism
- [P] Inorganic ion transport and metabolism
- [Q] Secondary metabolites biosynthesis, transport and catabolism

POORLY CHARACTERIZED
- [R] General function prediction only
- [S] Function unknown

Table S2. DART classification results for intra-species HGTs using different parameter settings. Results are shown for 36 different parameter setting combinations for number of phylogenetic neighbors, gene neighborhood size, additive HGT classification threshold, and replacing HGT classification threshold. Each cell shows 3 comma-separated values representing the number of additive, replacing, and ambiguous HGTs, respectively. The highlighted cell represents default parameter settings.

Additive threshold = 0%, Replacing threshold = 100%

	$m = 1$	$m = 2$	$m = 3$
$n = 4$	1445, 22785, 6957	770, 25326, 5091	542, 26486, 4159
$n = 8$	1288, 17355, 12544	707, 20681, 9799	495, 22330, 8362
$n = 16$	1168, 9618, 20401	698, 12555, 17934	484, 14363, 16340

Additive threshold $<$ 10%, Replacing threshold $>$ 90%

	$m = 1$	$m = 2$	$m = 3$
$n = 4$	1445, 22785, 6957	770, 25326, 5091	542, 26486, 4159
$n = 8$	1288, 17355, 12544	707, 20681, 9799	495, 22330, 8362
$n = 16$	1180, 16836, 13171	710, 19929, 10548	496, 21276, 9415

Additive threshold $<$ 20%, Replacing threshold $>$ 80%

	$m = 1$	$m = 2$	$m = 3$
$n = 4$	1445, 22785, 6957	770, 25326, 5091	542, 26486, 4159
$n = 8$	1304, 24157, 5726	723, 26364, 4100	511, 27174, 3502
$n = 16$	1202, 21709, 8276	730, 24050, 6407	512, 24873, 5802

Additive threshold $<$ 30%, Replacing threshold $>$ 70%

	$m = 1$	$m = 2$	$m = 3$
$n = 4$	1698, 27486, 2003	895, 28832, 1460	615, 29288, 1284
$n = 8$	1337, 26273, 3577	741, 27864, 2582	520, 28403, 2264
$n = 16$	1216, 22841, 7130	737, 24892, 5558	519, 25573, 5095

Table S3. Results of statistical analysis for inter-species additive HGTs as classified by DART. Results are shown for 36 different parameter setting combinations for DART, with the highlighted cell representing default parameter settings. Each cell shows 3 comma-separated values representing the percentage of inter-species additive, replacing, and ambiguous HGTs inferred by DART in the randomization analysis, respectively. The first value in each cell represents the estimated false-positive rate for inter-species additive HGTs classified by DART on the Aeromonas dataset. All results are averaged across 100 randomized runs.

Additive threshold = 0%, Replacing threshold = 100%

	$m=1$	$m=2$	$m=3$
$n=4$	1.78, 64.59, 33.63	0.91, 73.32, 25.77	0.59, 77.03, 22.38
$n=8$	1.38, 44.73, 53.89	0.65, 54.77, 44.58	0.59, 59.7, 39.71
$n=16$	1.3, 21.19, 77.51	0.69, 28.88, 70.43	0.47, 33.33, 66.2

Additive threshold $<$ 10%, Replacing threshold $>$ 90%

	$m=1$	$m=2$	$m=3$
$n=4$	1.72, 64.72, 33.56	0.85, 73.79, 25.36	0.58, 77.57, 21.85
$n=8$	1.34, 44.73, 53.93	0.74, 55.13, 44.13	0.42, 59.96, 39.62
$n=16$	1.95, 40.58, 57.47	1.01, 50.82, 48.17	0.69, 54.69, 44.62

Additive threshold $<$ 20%, Replacing threshold $>$ 80%

	$m=1$	$m=2$	$m=3$
$n=4$	1.67, 65.11, 33.22	0.84, 73.62, 25.54	0.63, 77.56, 21.81
$n=8$	2.11, 68.08, 29.81	1.17, 76.94, 21.89	**0.84**, 79.87, 19.29
$n=16$	3.31, 59.43, 37.26	1.63, 69.78, 28.59	1.23, 72.48, 26.29

Additive threshold $<$ 30%, Replacing threshold $>$ 70%

	$m=1$	$m=2$	$m=3$
$n=4$	4.24, 85.2, 10.56	2.15, 90.38, 7.47	1.62, 92.1, 6.28
$n=8$	3.44, 79.08, 17.48	1.66, 85.89, 12.45	1.32, 87.72, 10.96
$n=16$	4.52, 66.31, 29.17	2.06, 75.42, 22.52	1.78, 78.56, 19.66

Table S4. Results of statistical analysis for inter-species replacing HGTs as classified by DART. Results are shown for 36 different parameter setting combinations for DART, with the highlighted cell representing default parameter settings. Each cell shows 3 comma-separated values representing the percentage of inter-species additive, replacing, and ambiguous HGTs inferred by DART in the randomization analysis, respectively. The second value in each cell represents the estimated false-positive rate for inter-species replacing HGTs classified by DART on the Aeromonas dataset. All results are averaged across 100 randomized runs.

Additive threshold = 0%, Replacing threshold = 100%

	$m=1$	$m=2$	$m=3$
$n=4$	99.82, 0.01, 0.17	99.57, 0.03, 0.4	99.38, 0.02, 0.6
$n=8$	99.59, 0.01, 0.4	99.14, 0.02, 0.84	98.75, 0.03, 1.22
$n=16$	99.14, 0.01, 0.85	98.25, 0.01, 1.74	97.39, 0.01, 2.6

Additive threshold < 10%, Replacing threshold > 90%

	$m=1$	$m=2$	$m=3$
$n=4$	99.8, 0.02, 0.18	99.59, 0.02, 0.39	99.43, 0.02, 0.55
$n=8$	99.51, 0.01, 0.48	99.15, 0.02, 0.83	98.82, 0.02, 1.16
$n=16$	99.31, 0.02, 0.67	98.66, 0.01, 1.33	97.92, 0.02, 2.06

Additive threshold < 20%, Replacing threshold > 80%

	$m=1$	$m=2$	$m=3$
$n=4$	99.82, 0.01, 0.17	99.61, 0.02, 0.37	99.41, 0.04, 0.55
$n=8$	99.68, 0.02, 0.3	99.43, 0.04, 0.53	99.01, **0.04**, 0.95
$n=16$	99.46, 0.04, 0.5	98.86, 0.06, 1.08	98.4, 0.09, 1.51

Additive threshold < 30%, Replacing threshold > 70%

	$m=1$	$m=2$	$m=3$
$n=4$	99.94, 0.01, 0.05	99.81, 0.03, 0.16	99.71, 0.05, 0.24
$n=8$	99.8, 0.04, 0.16	99.6, 0.09, 0.31	99.37, 0.13, 0.5
$n=16$	99.57, 0.04, 0.39	99.21, 0.12, 0.67	98.76, 0.13, 1.11

Table S5. Results of statistical analysis for intra-species additive HGTs as classified by DART. Results are shown for 36 different parameter setting combinations for DART, with the highlighted cell representing default parameter settings. Each cell shows 3 comma-separated values representing the percentage of intra-species additive, replacing, and ambiguous HGTs inferred by DART in the randomization analysis, respectively. The first value in each cell represents the estimated false-positive rate for intra-species additive HGTs classified by DART on the Aeromonas dataset. All results are averaged across 100 randomized runs.

Additive threshold = 0%, Replacing threshold = 100%

	$m=1$	$m=2$	$m=3$
$n=4$	1.1, 73.27, 25.63	0.48, 81.41, 18.11	0.32, 84.81, 14.87
$n=8$	0.88, 56.22, 42.9	0.43, 66.27, 33.3	0.24, 71.48, 28.28
$n=16$	0.82, 31.36, 67.82	0.41, 40.38, 59.21	0.24, 46.48, 53.28

Additive threshold < 10%, Replacing threshold > 90%

	$m=1$	$m=2$	$m=3$
$n=4$	1.05, 73.71, 25.24	0.51, 81.29, 18.2	0.33, 84.8, 14.87
$n=8$	0.86, 56.35, 42.79	0.4, 66.14, 33.46	0.29, 71.44, 28.27
$n=16$	1.18, 54.86, 43.96	0.61, 64.41, 34.98	0.42, 68.64, 30.94

Additive threshold < 20%, Replacing threshold > 80%

	$m=1$	$m=2$	$m=3$
$n=4$	1.03, 73.71, 25.26	0.47, 81.39, 18.14	0.35, 85, 14.65
$n=8$	1.32, 78.62, 20.06	0.63, 85.23, 14.14	**0.44**, 87.67, 11.89
$n=16$	2.06, 71.68, 26.26	1.05, 79.12, 19.83	0.78, 81.61, 17.61

Additive threshold < 30%, Replacing threshold > 70%

	$m=1$	$m=2$	$m=3$
$n=4$	2.4, 90.23, 7.37	1.15, 93.72, 5.13	0.84, 94.85, 4.31
$n=8$	1.98, 86.51, 11.51	0.97, 90.79, 8.24	0.7, 92.1, 7.2
$n=16$	2.45, 76.93, 20.62	1.27, 83.2, 15.53	0.92, 85.52, 13.56

Table S6. Results of statistical analysis for intra-species replacing HGTs as classified by DART. Results are shown for 36 different parameter setting combinations for DART, with the highlighted cell representing default parameter settings. Each cell shows 3 comma-separated values representing the percentage of intra-species additive, replacing, and ambiguous HGTs inferred by DART in the randomization analysis, respectively. The second value in each cell represents the estimated false-positive rate for intra-species replacing HGTs classified by DART on the Aeromonas dataset. All results are averaged across 100 randomized runs.

Additive threshold = 0%, Replacing threshold = 100%

	$m = 1$	$m = 2$	$m = 3$
$n = 4$	99.8, 0.02, 0.18	99.57, 0.03, 0.4	99.4, 0.04, 0.56
$n = 8$	99.59, 0.01, 0.4	99.19, 0.02, 0.79	98.78, 0.04, 1.18
$n = 16$	99.16, 0.01, 0.83	98.32, 0.01, 1.67	97.51, 0.02, 2.47

Additive threshold $<$ 10%, Replacing threshold $>$ 90%

	$m = 1$	$m = 2$	$m = 3$
$n = 4$	99.82, 0.01, 0.17	99.6, 0.03, 0.37	99.38, 0.04, 0.58
$n = 8$	99.56, 0.01, 0.43	99.16, 0.02, 0.82	98.74, 0.03, 1.23
$n = 16$	99.32, 0.01, 0.67	98.68, 0.03, 1.29	97.92, 0.04, 2.04

Additive threshold $<$ 20%, Replacing threshold $>$ 80%

	$m = 1$	$m = 2$	$m = 3$
$n = 4$	99.81, 0.02, 0.17	99.61, 0.02, 0.37	99.39, 0.04, 0.57
$n = 8$	99.69, 0.02, 0.29	99.37, 0.03, 0.6	99.03, **0.06**, 0.91
$n = 16$	99.44, 0.04, 0.52	98.9, 0.08, 1.02	98.32, 0.13, 1.55

Additive threshold $<$ 30%, Replacing threshold $>$ 70%

	$m = 1$	$m = 2$	$m = 3$
$n = 4$	99.91, 0.02, 0.07	99.81, 0.04, 0.15	99.69, 0.06, 0.25
$n = 8$	99.77, 0.05, 0.18	99.56, 0.09, 0.35	99.29, 0.16, 0.55
$n = 16$	99.55, 0.06, 0.39	99, 0.15, 0.85	98.57, 0.21, 1.22

Table S7. Genomic context conservation results for unfiltered additive and replacing HGTs. For each category (row) of additive and replacing HGTs the table reports the percentage of HGTs that (i) have the same two flanking genes in both donor and recipient genomes, (ii) have at least one of the two flanking genes in common between donor and recipient, and (iii) have none of the two flanking genes in common between donor and recipient.

	**Both conserved**	**At least one conserved**	**No conservation**
**Intra-species additive**	71.2%	91.4%	8.6%
**Inter-species additive**	44.1%	76.9%	23.1%
**Phylo. distance ≥ 0.5 additive**	34.4%	70%	30%
**Intra-species replacing**	89.3%	99.5%	0.5%
**Inter-species replacing**	81.2%	97.8%	2.2%
**Phylo. distance ≥ 0.5 replacing**	71.5%	94.2%	5.8%

Table S8. Functional Enrichment in Additive and Replacing HGTs, shown separately for inter-species and intra-species HGTs. The table reports all COG functional categories that are enriched by a factor of at least 2 in either (i) inter-species replacing HGTs (top; middle column) and additive HGTs (bottom; middle column) or (ii) intra-species replacing HGTs (top; right column) and additive HGTs (bottom; right column). Reported enrichment factors are based on 4,384 inter-species additive and replacing HGTs and 19,790 intra-species additive and replacing HGTs that could be assigned to a proper COG functional category (i.e., not assigned to categories "S" or "#" in Supplemental Figures S5 and S6). Functional categories "A" and "B" were not considered since they were not well represented among the additive and replacing HGTs (fewer than 5 genes).

**Enriched in Replacing HGTs**		
**COG Functional Category**	**Inter-Species**	**Intra-Species**
[J] Translation, ribosomal structure and biogenesis	21.1 $\times$	∞ $\times$
[H] Coenzyme transport and metabolism	8.1 $\times$	4.7 $\times$
[O] Posttranslational modification, protein turnover, chaperone	4.7 $\times$	5.2 $\times$
[I] Lipid transport and metabolism	2.9 $\times$	∞ $\times$
[C] Energy production and conversion	2.8 $\times$	7.8 $\times$
[E] Amino acid transport and metabolism	2.3 $\times$	2.5 $\times$
[Q] Secondary metabolites biosynthesis, transport and catabolism	2.5 $\times$	1.7 $\times$
[P] Inorganic ion transport and metabolism	2.4 $\times$	1.3 $\times$
**Enriched in Additive HGTs**		
[L] Replication, recombination and repair	4.2 $\times$	4.4 $\times$
[V] Defense mechanisms	2.9 $\times$	1.8 $\times$
[M] Cell wall/membrane/envelope biogenesis	2.3 $\times$	3.1 $\times$
[K] Cell wall/membrane/envelope biogenesis	2.3 $\times$	1.5 $\times$
[N] Cell wall/membrane/envelope biogenesis	2.0 $\times$	1.5 $\times$

References

Colston S. M, Fullmer M. S, Beka L, Lamy B, Gogarten J. P and Graf J. 2014. Bioinformatic genome comparisons for taxonomic and phylogenetic assignments using Aeromonas as a test case. mBio **5**:e02136.

Gabler F, Nam S.-Z, Till S, Mirdita M, Steinegger M, Söding J, Lupas A. N and Alva V. 2020. Protein sequence analysis using the mpi bioinformatics toolkit. Current Protocols in Bioinformatics **72**:e108.

Krumsiek J, Arnold R and Rattei T. 2007. Gepard: a rapid and sensitive tool for creating dotplots on genome scale. Bioinformatics (Oxford, England) **23**:1026–1028.

Neuwirth E. 2014. RColorBrewer: ColorBrewer Palettes. URL `https://CRAN.R-project.org/package=RColorBrewer`.

Pearson W. R. 1996. Effective protein sequence comparison. In Computer Methods for Macromolecular Sequence Analysis, vol. 266 of *Methods in Enzymology*, 227–258. Academic Press.

Seemann T. 2014. Prokka: rapid prokaryotic genome annotation. Bioinformatics (Oxford, England) **30**:2068–2069.

Tatusov R. L, Galperin M. Y, Natale D. A and Koonin E. V. 2000. The COG database: a tool for genome-scale analysis of protein functions and evolution. Nucleic Acids Research **28**:33–36.

Wickham H. 2016. ggplot2: Elegant Graphics for Data Analysis. Springer-Verlag New York. URL `https://ggplot2.tidyverse.org`.

Wilkins D and Kurtz Z. 2020. gggenes: Draw Gene Arrow Maps in 'ggplot2'. URL `https://CRAN.R-project.org/package=gggenes`.

Yutani H. 2021. gghighlight: Highlight Lines and Points in 'ggplot2'. URL `https://CRAN.R-project.org/package=gghighlight`.
